# Serum C-C motif chemokine ligand 17 as a predictive biomarker for the progression of non-idiopathic pulmonary fibrosis interstitial lung disease

**DOI:** 10.1186/s12931-025-03237-2

**Published:** 2025-04-23

**Authors:** Takatoshi Enomoto, Yoshito Takeda, Yuya Shirai, Takehiro Hasegawa, Feng Zhao, Hanna Lunding, Moritz Pohl, Ryuya Edahiro, Shigeyuki Shichino, Takahiro Kawasaki, Hanako Yoshimura, Reina Hara, Saori Amiya, Makoto Yamamoto, Daisuke Nakatsubo, Satoshi Tanizaki, Mana Nakayama, Yoshimi Noda, Takayuki Niitsu, Yuichi Adachi, Mari Tone, Yuko Abe, Maiko Naito, Kentaro Masuhiro, Yujiro Naito, Takayuki Shiroyama, Kotaro Miyake, Shohei Koyama, Kiyoharu Fukushima, Kota Iwahori, Haruhiko Hirata, Izumi Nagatomo, Satoshi Nojima, Masahiro Yanagawa, Yoshikazu Inoue, Atsushi Kumanogoh

**Affiliations:** 1 Department of Respiratory Medicine and Clinical Immunology, Osaka University Graduate School of Medicine, Suita, Osaka Japan; 2 Department of Statistical Genetics, Osaka University Graduate School of Medicine, Suita, Osaka Japan; 3 Research and Development Division, Sysmex R&D Center Europe GmbH, Hamburg, Germany; 4 Research and Development Division, Sysmex R&D Centre UK, Cambridge, UK; 5 Laboratory for Systems Genetics, RIKEN Center for Integrative Medical Sciences, Yokohama, Japan; 6 Division of Molecular Regulation of Inflammatory and Immune Diseases, Research Institute of Biomedical Sciences, Tokyo University of Science, Chiba, Japan; 7 Department of Pathology, Osaka University Graduate School of Medicine, Suita, Osaka Japan; 8 Department of Radiology, Osaka University Graduate School of Medicine, Suita, Osaka Japan; 9 Clinical Research Center, NHO Kinki Chuo Chest Medical Center, Sakai, Osaka Japan; 10 Osaka Anti-Tuberculosis Association, Osaka Fukujuji Hospital, Neyagawa, Osaka Japan; 11 Center for Infectious Diseases for Education and Research (CiDER), Osaka University, Suita, Osaka Japan; 12 Integrated Frontier Research for Medical Science Division, Institute for Open and Transdisciplinary Research Initiatives (OTRI), Osaka University, Suita, Osaka Japan; 13 Department of Immunopathology, Immunology Frontier Research Center (WPI-IFReC), Osaka University, Suita, Osaka Japan; 14 Center for Advanced Modalities and DDS (CAMaD), Osaka University, Osaka, Japan; 15 Department of Respiratory Medicine and Clinical Immunology, Graduate School of Medicine, Osaka University, 2-2 Yamadaoka, Suita, Osaka 565-0871 Japan

**Keywords:** Lung diseases, Interstitial, Fibrosis, Prognosis, Pulmonary surfactant-associated protein B, Periostin

## Abstract

**Background:**

Interstitial lung disease (ILD), represented by idiopathic pulmonary fibrosis (IPF) and progressive pulmonary fibrosis (PPF), shows poor prognosis due to progressive fibrosis. Early therapeutic intervention is required to enhance the efficacy of antifibrotic drugs, highlighting the importance of early detection of ILD progression. Although candidate biomarkers for predicting ILD progression have been recently reported through omics analyses, clinically measurable biomarkers remain unestablished. This study aimed to identify clinically measurable biomarkers that could predict the degree of ILD progression.

**Methods:**

The serum levels of 13 candidate biomarkers were prospectively measured by chemiluminescent enzyme immunoassay and the utilities for predicting ILD progression were compared in the discovery cohort (total 252 patients). Moreover, we evaluated the utility of the identified biomarker in another independent cohort (154 patients with non-IPF-ILD) and examined the dynamics of the biomarker by immunoblotting and single-cell RNA sequencing (scRNA-seq) using samples of patients and a mouse model.

**Results:**

In the discovery cohort, C-C motif chemokine ligand (CCL)17 could reliably predict ILD progression, particularly in patients with ILD other than IPF, and showed significant associations with mortality (hazard ratio [HR] 3.70; 95% confidence interval [CI] 1.19–11.49; *P* = 0.015; cut-off value = 418 pg/mL). Consistently, in the validation cohort, the CCL17 high group showed significantly higher mortality (HR: 2.15; 95% CI 0.99–4.69; *P* = 0.049), and CCL17 was identified as an independent prognostic factor from corticosteroid or immunosuppressive agents use and ILD-gender-age-physiology index. Similar to the results of serum studies, CCL17 levels in the lungs of patients with PPF and model mice were higher than those in controls. They were positively correlated with CCL17 levels in the serum, suggesting that the increased serum CCL17 levels could reflect an increase in CCL17 levels in lung tissues. The scRNA-seq analysis of lung tissues from model mice suggested that the levels of CCL17 derived primarily from conventional dendritic cells and macrophages increased, especially during the profibrotic phase.

**Conclusions:**

We identified serum CCL17 as a clinically measurable biomarker for predicting non-IPF-ILD progression. Serum CCL17 could enable the stratification of patients at risk of non-IPF-ILD progression, leading to appropriate early therapeutic intervention.

**Graphical abstract:**

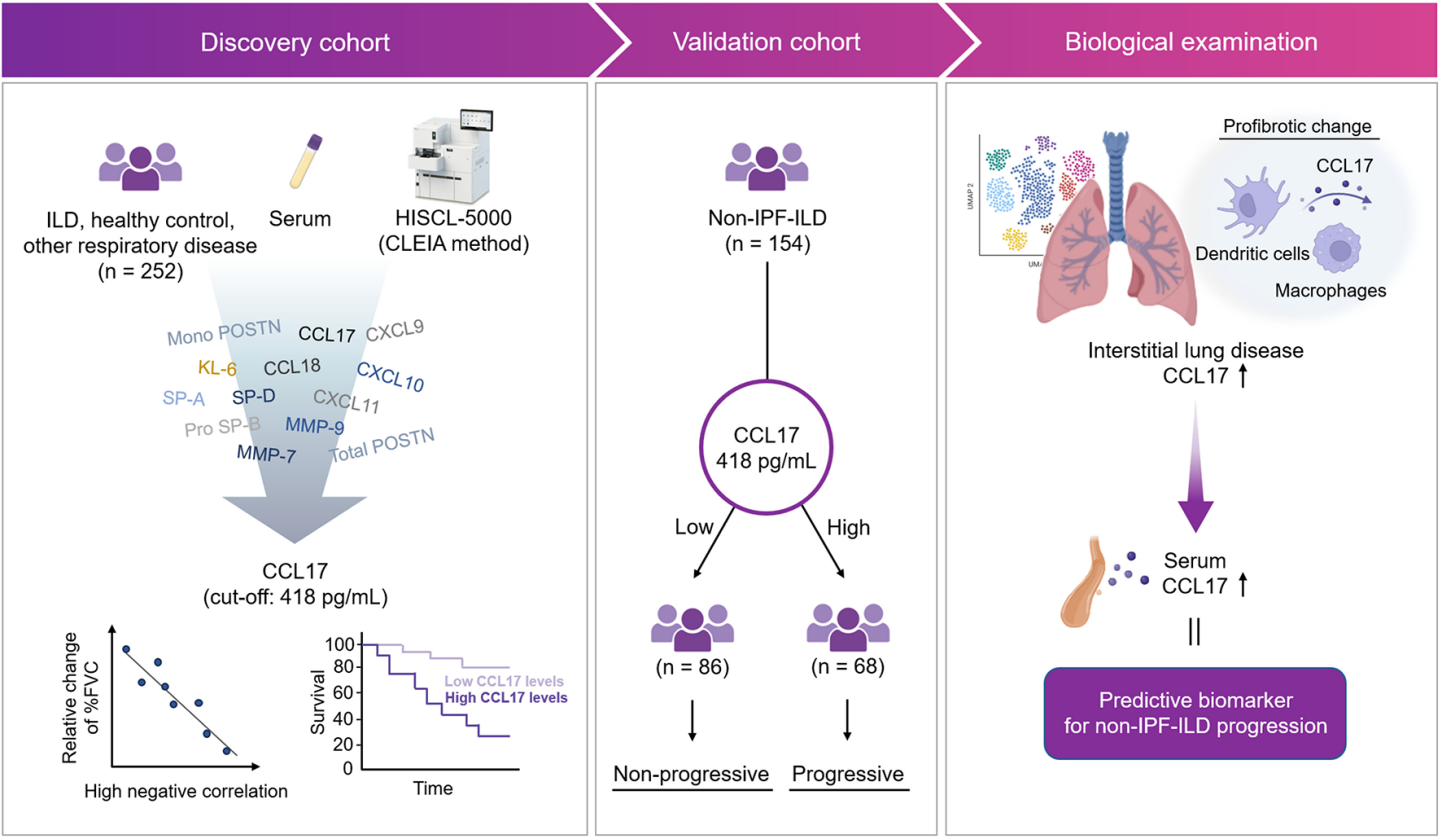

**Supplementary Information:**

The online version contains supplementary material available at 10.1186/s12931-025-03237-2.

## Background

Interstitial lung diseases (ILDs), characterized by varying degrees of inflammation and fibrosis, are a heterogeneous group of parenchymal pulmonary disorders [1, 2]. Idiopathic pulmonary fibrosis (IPF) and progressive pulmonary fibrosis (PPF) exhibit progressive fibrosis, which leads to a decline in lung function and early death [1]. Antifibrotic drugs are effective in treating these diseases [3–5]; however, early therapeutic intervention is required to facilitate better efficacy [6, 7], thereby emphasizing the value of the early detection of the ILD progression.

Based on recent progress in next-generation sequencing and mass spectrometry, several candidate biomarkers associated with ILD progression have been identified via comprehensive omics analysis [8–10]. Additionally, by focusing on serum extracellular vesicles (EVs), we have previously demonstrated that pro-surfactant protein [SP]-B is associated with non-IPF-ILD progression [11]. However, it remains unclear whether these biomarkers exhibit utilities in a clinically applicable measurement system.

Thus, we sought to identify clinically measurable biomarkers that could predict the degree of ILD progression. First, we prospectively measured the levels of 13 candidate biomarkers based on previous reports [8, 9, 11–14], via chemiluminescent enzyme immunoassay (CLEIA), a clinically applicable assay system and compared the utility for predicting ILD progression in the discovery cohort. Thereafter, we set a specific cut-off value for the biomarker that showed the best predictive performance and evaluated the reproducibility of the utility further in another independent cohort. Finally, we examined the dynamics of the biomarker during the development of pulmonary fibrosis using samples from patients with PPF and a bleomycin-induced pulmonary fibrosis mouse model.

## Methods

Methodological details are available in Supplementary methods.

### Study population

In the discovery cohort, we reviewed cases included in the dataset from our previous study [11], focusing on those with available cryopreserved serum samples. Patients with ILD experiencing acute exacerbations, bacterial infection, or any other unstable medical condition at the time of blood collection were excluded. Thus, the discovery cohort comprised 143 patients with ILD, categorized into IPF [*n* = 86] and non-IPF-ILD [*n* = 57], along with 30 healthy controls and 79 patients diagnosed with other respiratory diseases (including bronchial asthma [*n* = 30], chronic obstructive pulmonary disease [COPD; *n* = 28], nontuberculous mycobacterial infection [*n* = 15], and bacterial pneumonia [*n* = 6]).

The inclusion criteria for the validation cohort were as follows: (i) inclusion in our serum sample database and (ii) diagnosis of non-IPF-ILD. The 7607 serum samples collected between May 23, 2012 and February 10, 2023 and stored at − 80 °C at the Osaka University Hospital were reviewed. Our cryopreserved serum samples include both those taken at diagnosis and those taken during the advanced stages of the disease. If there were serum samples collected at different times originating from the same patient, the serum sample collected earliest was adopted. Consequently, 331 patients with non-IPF-ILD were identified. The exclusion criteria were as follows: (i) inclusion in the discovery cohort, (ii) cryopreserved serum samples not available, (iii) acute exacerbation, bacterial infection, or any other unstable medical condition, (iv) eosinophilic disease such as asthma and atopic dermatitis or increased blood eosinophil counts (> 500/µl), (v) diagnosis other than idiopathic nonspecific interstitial pneumonia (NSIP), connective tissue disease (CTD)-ILD, unclassifiable ILD, or fibrotic hypersensitivity pneumonitis (FHP). Here, to reduce the prognostic impact of ILD heterogeneity [15], we limited our analysis to the patients with ILD including idiopathic NSIP, CTD-ILD, FHP, and unclassifiable ILD [15]. Thus, 154 consecutive patients with non-IPF-ILD were included in the validation cohort. Subsequently, the levels of the identified biomarker in the cryopreserved serum samples were measured and the enrolled patients were assigned to groups with high and low levels of the biomarker (Fig. 4).

### Assessment of ILDs

All ILD cases were diagnosed through multidisciplinary discussion based on the American Thoracic Society and European Respiratory Society guidelines [1, 16]. The diagnosis of idiopathic NSIP in patients without surgical lung biopsy was based on the absence of underlying disease and the NSIP pattern on CT images. The ILD-gender-age-physiology (GAP) index was calculated according to the definition by Ryerson et al. [15]. ILD progression was defined as > 10% of relative decline in percent predicted forced vital capacity (%FVC), acute exacerbation, or death within a year.

### Measurement of the serum levels of 13 candidate biomarkers using CLEIA

In the discovery cohort, serum levels of 13 candidate biomarkers (pro SP-B, SP-A, SP-D, Krebs von den Lungen-6 [KL-6], monomeric periostin [mono POSTN], total POSTN, C-C motif chemokine ligand [CCL]17, CCL18, C-X-C motif chemokine ligand [CXCL]-9, CXCL10, CXCL11, matrix metalloproteinase [MMP]-7, and MMP9) were measured at Sysmex Corporation by CLEIA using HISCL-5000™ (Sysmex Corporation, Kobe, Japan) (Supplementary methods, Supplementary Fig. S1, and Supplementary Table S1 and S2).

In the validation cohort, the serum levels of CCL17 were commercially measured at BML Inc. by CLEIA using HISCL-5000™, as well as the discovery cohort. We confirmed an extremely strong correlation between serum CCL17 levels measured by the assay systems used in the two cohorts (Supplementary Fig. S2).

### Outcomes in the discovery and validation cohorts

In the discovery cohort, we evaluated the followings: (i) serum levels of the 13 candidate biomarkers for each disease, (ii) candidate biomarker levels of patients with and without ILD progression, (iii) performance of candidate biomarkers for predicting ILD progression by receiver operating characteristic (ROC) analysis, (iv) correlation between relative change of %FVC at 1 year and the candidate biomarker levels, and (v) overall survival (OS) in groups with high and low levels of each candidate biomarker. Moreover, for the biomarker that showed the highest performance for predicting ILD progression, a cut-off value was defined, and the percentages of patients with ILD progression within 1 year in groups with high and low levels of the biomarker were evaluated.

In the validation cohort, the primary outcome was OS during 5 years. The secondary outcomes were as follows: (i) relative change of %FVC at 1 year, (ii) ROC analysis for evaluating the biomarker as predicting ILD progression (iii) percentage of patients with ILD progression within 1 year, and (iv) subgroup analysis of percentage of patients with ILD progression within 1 year, by ILD classification.

### Statistical analyses

The serum levels of the candidate biomarkers were compared based on conditions using analysis of variance (ANOVA), and the Dunnett’s method was applied to adjust the obtained ANOVA *P* values.

Differences in the candidate biomarker levels between patients with and without ILD progression were compared using the Mann–Whitney U test and adjusted using the Bonferroni’s method.

ROC analysis was performed to evaluate the efficacy of candidate biomarkers for predicting ILD progression.

The correlation between the candidate biomarker levels and the relative change of %FVC at 1 year was analyzed using Spearman’s correlation analysis.

OS was estimated using the Kaplan–Meier method, and the log-rank test was used to assess differences between two comparison groups. Univariable and multivariable Cox proportional hazard regression models were adopted to determine hazard ratios (HRs). In the Cox proportional hazard regression models, it was confirmed that the proportional hazards assumption was maintained. OS was defined as the period from the date of blood collection to the date of death from any cause. In the validation cohort, OS was set for a maximum of five years. Data for patients not reported as deceased at the time of analysis were censored on the date they were last known to be alive.

Differences in the relative change of %FVC at 1 year between groups with high and low levels of the biomarker were compared using the Mann–Whitney U test.

Differences in the percentage of patients with ILD progression within 1 year between groups with high and low levels of the biomarker were compared using Fisher’s exact tests.

The final analysis was conducted on July 10, 2023, for the discovery cohort and on February 8, 2024, for the validation cohort using the patients’ medical records. In each analysis, cases without the necessary data were excluded.

The above statistical analyses were performed using the EZR software, version 1.38. Statistical significance was set at *P* < 0.05.

## Results

### Participant demographics in the discovery cohort

Table 1 and Supplementary Table S3 present the baseline characteristics at the time of blood collection.

**Table 1:** Baseline characteristics at the time of blood collection of the discovery cohort

	HC^*^	BA^†^	COPD^‡^	NTM^§^	Bacterial pneumonia	IPF^ll^	Non-IPF-ILD^**^
No. of subjects	30	30	28	15	6	86	57
Age (years)	62 (55–73)	67 (48–76)	77 (73–80)	71 (63–78)	68 (59–69)	75 (70–79)	73 (65–76)
Sex (male)	10 (33%)	8 (27%)	24 (86%)	1 (7%)	3 (50%)	66 (77%)	29 (51%)
Smoking history (yes)	1 (3%)	5 (17%)	27 (96%)	0 (0%)	2 (33%)	62 (72%)	30 (53%)
Corticosteroid (yes)	0 (0%)	5 (17%)	0 (0%)	1 (7%)	0 (%)	12 (14%)	22 (39%)
Immunosuppressive agents (yes)	0 (0%)	0 (0%)	1 (4%)	2 (13%)	2 (33%)	3 (3%)	11 (19%)
Antifibrotic therapy (yes)	0 (0%)	0 (0%)	0 (0%)	0 (0%)	0 (0%)	18 (21%)	1 (2%)
Serum biomarker							

CCL17^‡‡^ (pg/mL)	230 (194–289)	335 (181–454)	270 (202–389)	259 (164–357)	209 (134–262)	515 (343–767)	418 (330–642)
Classification of ILD							
INSIP^§§^	N/A	N/A	N/A	N/A	N/A	N/A	14 (25%)
CTD-ILD^llll^	N/A	N/A	N/A	N/A	N/A	N/A	24 (42%)
Unclassifiable ILD	N/A	N/A	N/A	N/A	N/A	N/A	15 (26%)
FHP^***^	N/A	N/A	N/A	N/A	N/A	N/A	1 (2%)
PPFE^†††^	N/A	N/A	N/A	N/A	N/A	N/A	3 (5%)

Data are presented as the median (interquartile range) or number of patients (percentage)

^*^healthy control; ^†^bronchial asthma; ^‡^chronic obstructive pulmonary disease; ^§^nontuberculous mycobacterial infection; ^ll^idiopathic pulmonary fibrosis; ^**^interstitial lung disease; ^††^not available; ^‡‡^C-C motif chemokine ligand 17; ^§§^idiopathic nonspecific interstitial pneumonia; ^llll^connective tissue disease-interstitial lung disease; ^***^fibrotic hypersensitivity pneumonitis; ^†††^pleuroparenchymal fibroelastosis

### Specificity of the 13 candidate biomarkers for detecting ILD

**Fig. 1:**
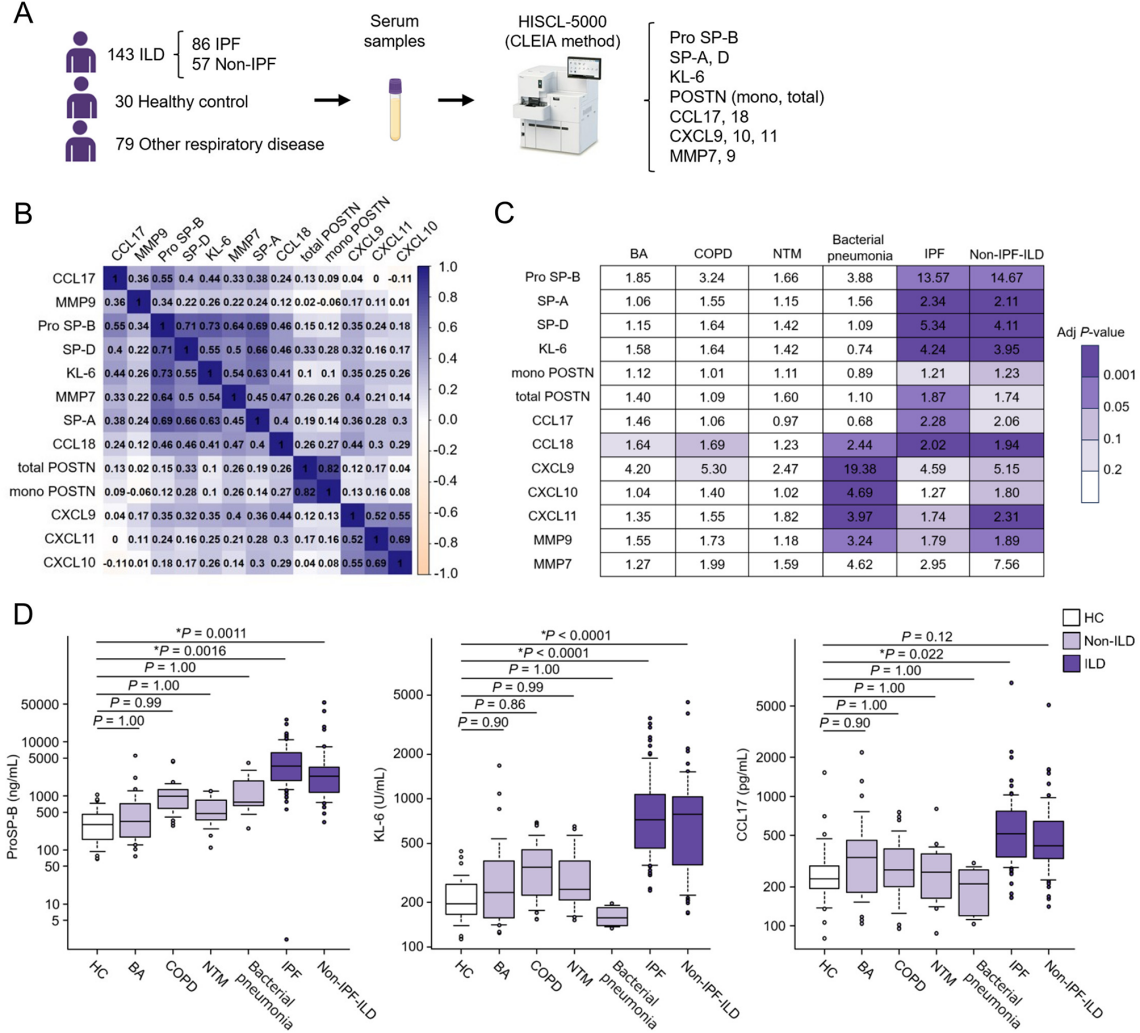
Evaluation of specificity of the 13 candidate biomarkers for detecting ILD. (**A**) Overview of the study design. (**B**) Correlation matrix analysis of the 13 candidate biomarkers. The values in the heatmap represent Spearman’s correlation coefficients. (**C**, **D**) Examination of ILD specificity of 13 candidate biomarkers and excerpts on pro SP-B, KL-6, and CCL17. The values on the heatmap represent fold change compared with that in HCs. The serum levels of the candidate biomarkers were compared using analysis of variance (ANOVA), and the Dunnett’s method was applied to adjust the *P* values obtained via ANOVA. *, adjusted *P* < 0.05. Numbers of samples: HC (*n* = 30), BA (*n* = 30), COPD (*n* = 28), NTM (*n* = 15), bacterial pneumonia (*n* = 6), IPF (*n* = 86), non-IPF-ILD (*n* = 57). BA, bronchial asthma; CCL, C-C motif chemokine ligand; COPD, chronic obstructive pulmonary disease; HC, healthy control; ILD, interstitial lung disease; IPF, idiopathic pulmonary fibrosis; KL-6, Krebs von den Lungen-6; NTM, nontuberculous mycobacterial infection; SP-B, surfactant protein-B

Serum levels of the 13 candidate biomarkers were measured using CLEIA (Fig. 1A). Correlation matrix analysis showed that proSP-B, SP-A, SP-D, KL-6, and MMP7; mono POSTN and total POSTN; and CXCL9, CXCL10, and CXCL11 were strongly correlated with each other (Fig. 1B). The serum levels of all candidate biomarkers, except those of MMP7, were elevated in patients with ILD compared with those in healthy controls. The CCL18, CXCL9, CXCL10, CXCL11, and MMP9 levels were also elevated in patients with other respiratory diseases, such as bacterial pneumonia and COPD. The pro-SP-B, SP-A, SP-D, KL-6, POSTN, and CCL17 levels exhibited ILD-specific increases (Fig. 1C). Figure 1D present the serum levels of pro SP-B, KL-6, and CCL17 in each disease group, respectively.

### Utility of the 13 candidate biomarkers for predicting ILD progression

**Fig. 2:**
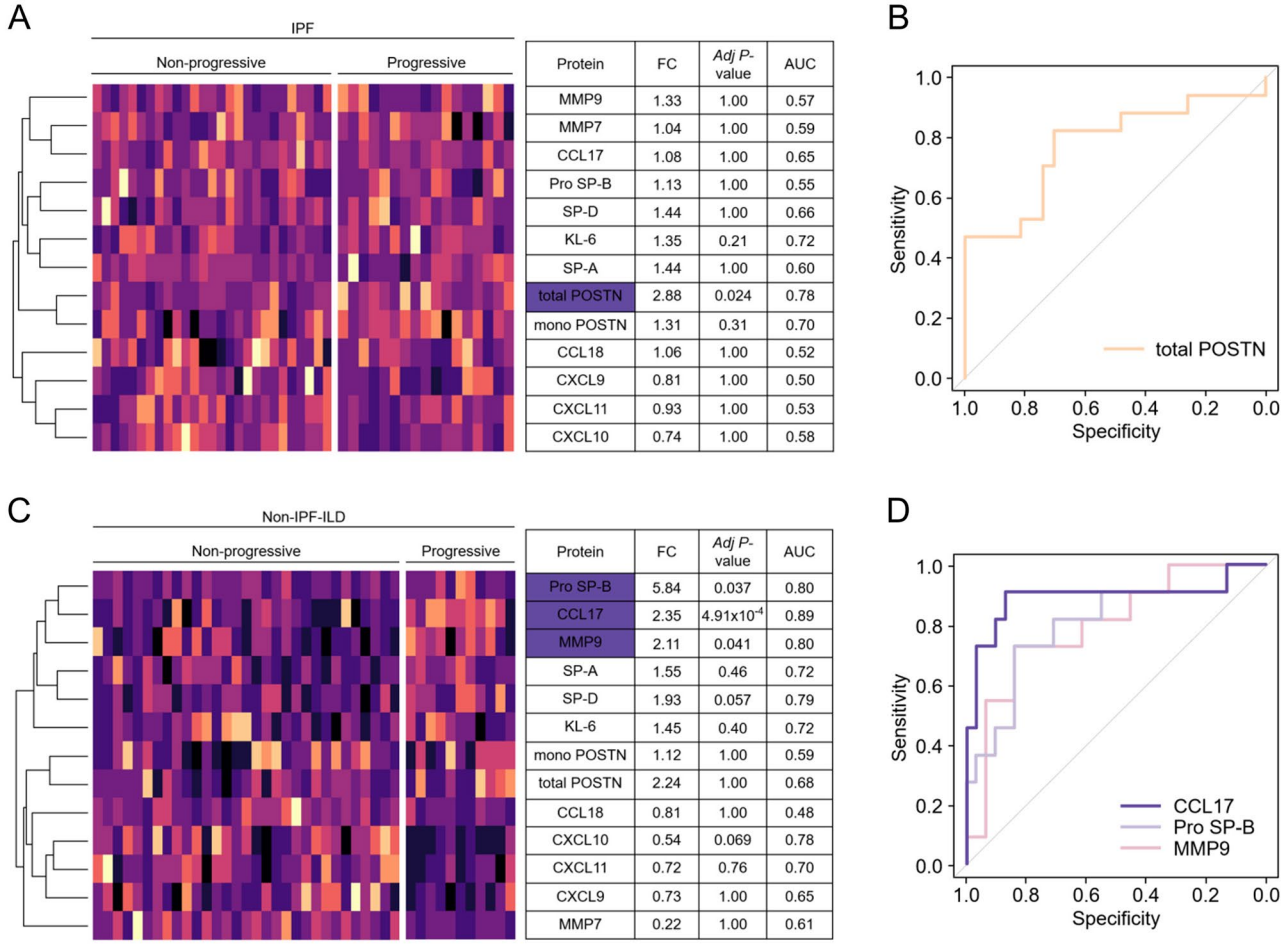
Evaluation of utility of the 13 candidate biomarkers for predicting ILD progression. (**A**, **B**) Heatmap and diagnostic values of 13 candidate biomarkers for predicting ILD progression in patients with IPF and excerpts on total POSTN. (**C**, **D**) The heatmap and diagnostic values of 13 candidate biomarkers for predicting ILD progression in patients with non-IPF-ILD and excerpts on CCL17. ILD progression was defined as > 10% of relative decline in %FVC, acute exacerbation, or death within a year. Differences between the candidate biomarker levels of patients with and without ILD progression were compared using the Mann–Whitney U test. The proteins marked in blue exhibited significant variability after adjustment with Bonferroni’s method. CCL, C-C motif chemokine ligand; ILD, interstitial lung disease; IPF, idiopathic pulmonary fibrosis; POSTN, periostin

The serum levels of the 13 candidate biomarkers in patients with ILD who did and did not exhibit progression within 1 year were compared to identify biomarkers associated with future ILD progression. The total POSTN levels were significantly elevated in the progressive IPF group compared with the non-progressive IPF group (Bonferroni’s adjusted *P* = 0.024) (Fig. 2A). ROC curve analysis yielded an area under the curve (AUC) of 0.78 (95% confidence interval [CI] 0.62–0.93) (Fig. 2B). The pro-SP-B, CCL17, and MMP9 levels were significantly elevated in the progressive non-IPF-ILD group compared with the non-progressive non-IPF-ILD group (Bonferroni’s adjusted *P* = 0.038, 0.00049, and 0.042, respectively) (Fig. 2C). ROC curve analysis revealed that the CCL17 levels exhibited the best performance (AUC 0.89; 95% CI 0.74–1.00) and were superior to the ILD-GAP index (AUC 0.81; 95% CI 0.66–0.95) (Fig. 2D). Pro SP-B levels exhibited the second best performance (AUC 0.80, 95% CI 0.63–0.97). Among combinations of any two candidate biomarkers, the combination of CCL17 and total POSTN showed the best performance (AUC 0.98, 95% CI 0.95–1.00) (Supplementary Fig. S3).

**Fig. 3:**
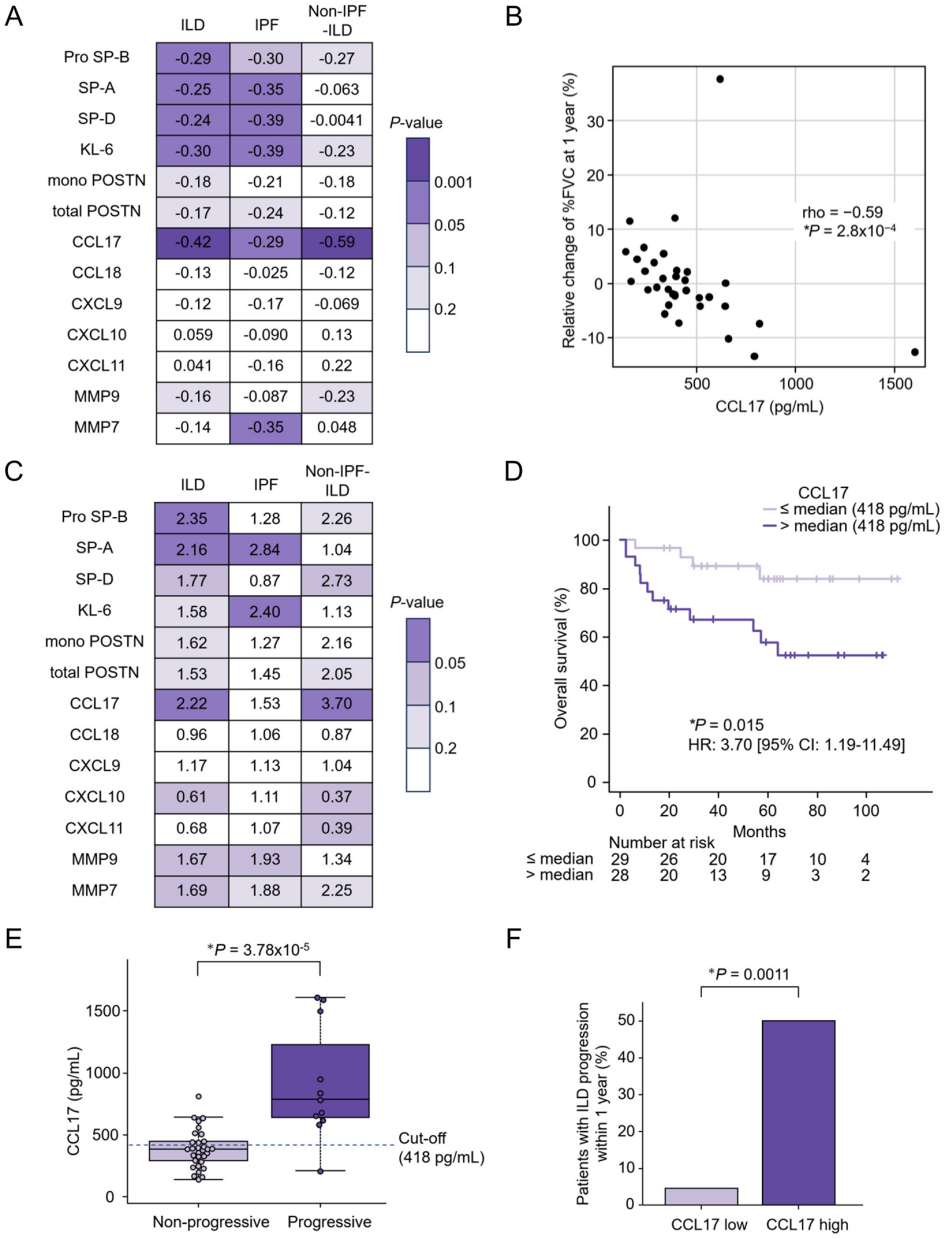
Utility of serum CCL17 levels for predicting non-IPF-ILD progression in the discovery cohort. (**A**, **B**) Examination of the correlations between 13 candidate biomarkers and relative change of %FVC at 1 year and excerpts on CCL17. The relationship was analyzed using Spearman’s correlation analysis. The values in the heatmap represent the rho values. *, *P* < 0.05. Numbers of samples: interstitial lung disease (ILD; *n* = 72), idiopathic pulmonary fibrosis (IPF; *n* = 38), non-IPF-ILD (*n* = 34). (**C**, **D**) Examination of the correlations of the 13 candidate biomarkers with prognosis and excerpts on CCL17. Overall survival was estimated using the Kaplan–Meier method, and the log-rank test was used to assess the differences between the two comparison groups (serum levels > median vs. ≤ median). Univariate Cox proportional hazard regression models were used to determine the HRs of the two comparison groups (serum levels > median or ≤ median). The values in the heatmap represent HR values. *, *P* < 0.05. Numbers of samples: ILD (*n* = 143), IPF (*n* = 86), non-IPF-ILD (*n* = 57). (**E**) Serum CCL17 levels of patients with and without ILD progression within 1 year for each disease. (**F**) The percentages of patients with ILD progression within 1 year in groups with high and low CCL17 levels for each disease. (**E**, **F**) Differences between both groups were compared using the Mann–Whitney U test and Fisher’s exact tests, respectively. CCL, C-C motif chemokine ligand; %FVC, percent predicted forced vital capacity; HR, hazard ratio; ILD, interstitial lung disease; IPF, idiopathic pulmonary fibrosis

CCL17 has consistently shown the most significant negative correlation with the relative change in %FVC at 1 year in patients with ILD (rho = -0.42, *P* = 2.79 × 10^− 4^) and especially in those with non-IPF-ILD (rho = -0.59, *P* = 2.80 × 10^− 4^) (Fig. 3A and B). Univariate analysis by Cox proportional hazards analysis and log-rank tests (> median vs. ≤ median) revealed that serum CCL17 levels exhibited the most significant associations with mortality in patients with non-IPF-ILD (> median [= 418 pg/mL] vs. ≤ median; HR 3.70; 95% CI 1.19–11.49; *P* = 0.015 by log-rank test) (Fig. 3C and D). Serum pro SP-B levels exhibited the most significant associations with mortality in patients with ILD (HR 2.35; 95% CI 1.27–4.36; *P* = 0.0051 by log-rank test), although no significant association was found in patients with IPF (HR 1.28; 95% CI 0.60–2.73; *P* = 0.53 by log-rank test) and in patients with non-IPF-ILD (HR 2.26; 95% CI 0.82–6.25; *P* = 0.11 by log-rank test) (Supplementary Fig. S4A–C).

### Setting the cut-off value for serum CCL17 levels as a predictive biomarker for non-IPF-ILD progression

Thus, we found that serum CCL17 was prominently associated with non-IPF-ILD progression. Considering the remarkable results of the univariate analysis by Cox proportional hazards model and log-rank tests (> median vs. ≤ median) in patients with non-IPF-ILD, we set the cut-off value for CCL17 at 418 pg/mL. In that case, the percentage of patients with non-IPF-ILD who exhibited progression within 1 year was significantly higher in the CCL17 high group than in the CCL17 low group (50.00% vs. 4.55%; *P* = 0.0011) (Fig. 3E and F).

### Participant demographics in the validation cohort

**Fig. 4:**
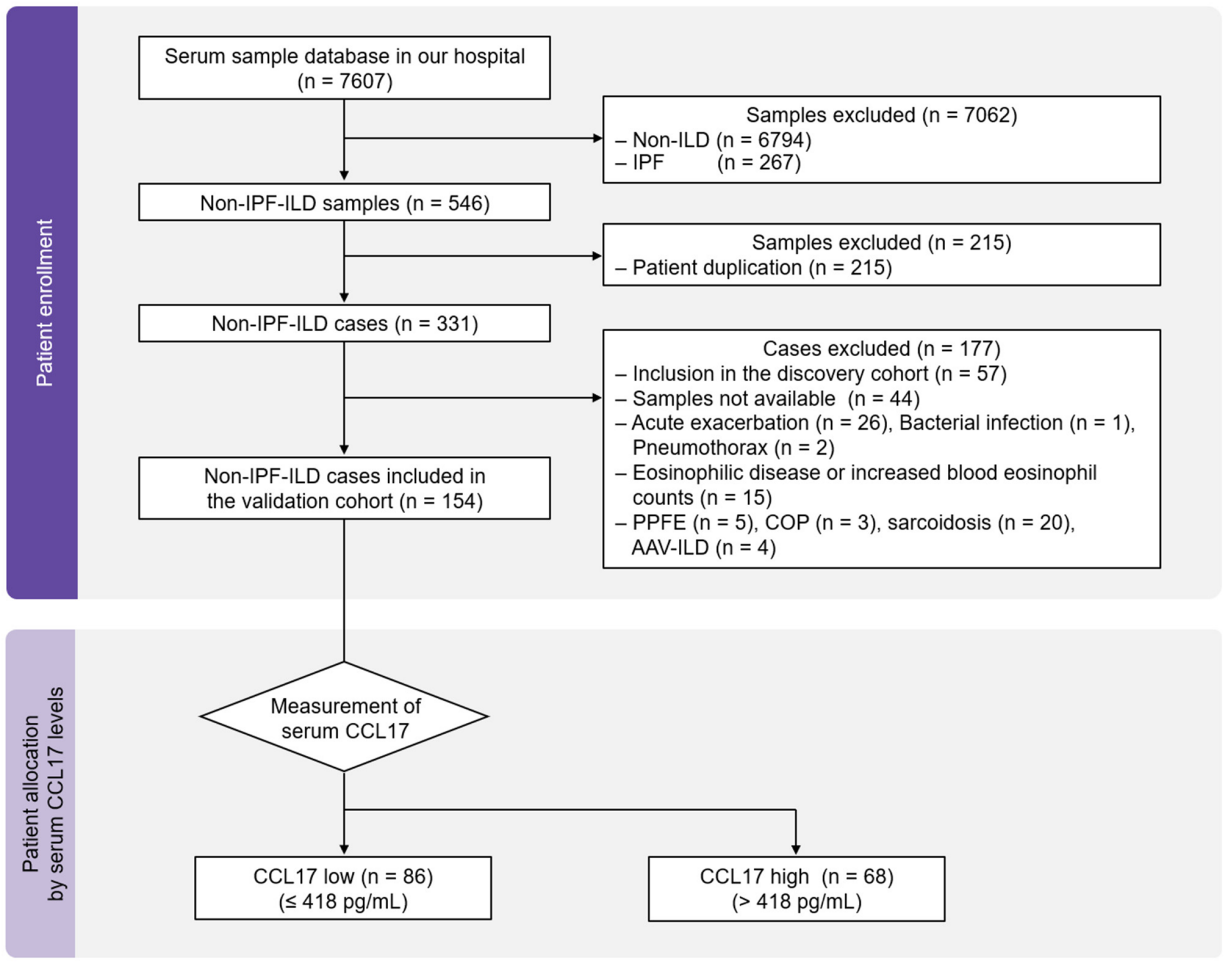
Patient enrollment and allocation in the validation cohort. AAV, anti-neutrophil cytoplasmic antibody-associated vasculitis; CCL, C-C motif chemokine ligand; COP, cryptogenic organizing pneumonia; ILD, interstitial lung disease; IPF, idiopathic pulmonary fibrosis; PPFE, pleuroparenchymal fibroelastosis

To evaluate the reproducibility of the utility of CCL17 (cut-off: 418 pg/mL) for predicting non-IPF-ILD progression, the validation cohort was conducted. Serum CCL17 levels of the 154 enrolled patients were measured and they were assigned to the CCL17 high (*n* = 68) and low (*n* = 86) groups (Fig. 4). Table 2 present the baseline characteristics at the time of blood collection. Compared to the CCL17 low group, the CCL17 high group showed lower %FVC and %DLco. However, the other baseline characteristics in the two groups, including ILD-GAP index, were well balanced.

**Table 2:** Baseline characteristics at the time of blood collection of the validation cohort

	Non-IPF-ILD^‡^		
	CCL17 low	CCL17 high	*P* value
No. of subjects	86	68	-
Age (years)	72 (67–76)	71 (62–77)	0.55
Sex (male)	42 (49%)	39 (57%)	0.33
Smoking history (yes)	45 (52%)	35 (51%)	1.00
Corticosteroid (yes)	21 (24%)	24 (35%)	0.16
Immunosuppressive agents (yes)	11 (13%)	11 (16%)	0.65
Antifibrotic therapy (yes)	2 (2.3%)	2 (2.9%)	1.00
Classification of ILD			
INSIP^‡‡^	26 (30%)	31 (46%)	0.065
CTD-ILD^§§^	30 (35%)	17 (25%)	0.22
Unclassifiable ILD	29 (34%)	15 (22%)	0.15
FHP^llll^	1 (1%)	5 (7%)	0.088
Pulmonary function tests			
%FVC^‡‡‡^	88.2 (73.2–101.0)	74.1 (59.8–89.9)	*0.00070
%DLco^§§§^	70.6 (46.7–84.4)	57.2 (37.3–72.2)	*0.0081
ILD-GAP^llllll^ index			0.83
0–1	39 (45%)	29 (43%)	-
2–3	31 (36%)	28 (41%)	-
4–5	6 (7%)	6 (9%)	-
6–8	1 (1%)	2 (3%)	-
N/A	9 (10%)	3 (4%)	-
Follow up time (months)	59.7 (33.5–60.0)	34.5 (20.1–60.0)	*0.0049

Data are presented as the median (interquartile range) or number of patients (percentage). Differences in the two groups were assessed by the Mann-Whitney U test or Fisher’s exact test. *, *P* < 0.05

^†^idiopathic pulmonary fibrosis; ^‡^interstitial lung disease; ^§^not available; ^ll^total periostin; ^††^C-C motif chemokine ligand 17; ^‡‡^idiopathic nonspecific interstitial pneumonia; ^§§^connective tissue disease-interstitial lung disease; ^llll^fibrotic hypersensitivity pneumonitis; ^†††^pleuroparenchymal fibroelastosis; ^‡‡‡^%forced vital capacity; ^§§§^%diffusing capacity for carbon monoxide; ^llllll^gender age physiology

### Reproduction of the utility of serum CCL17 levels for predicting non-IPF-ILD progression

**Fig. 5:**
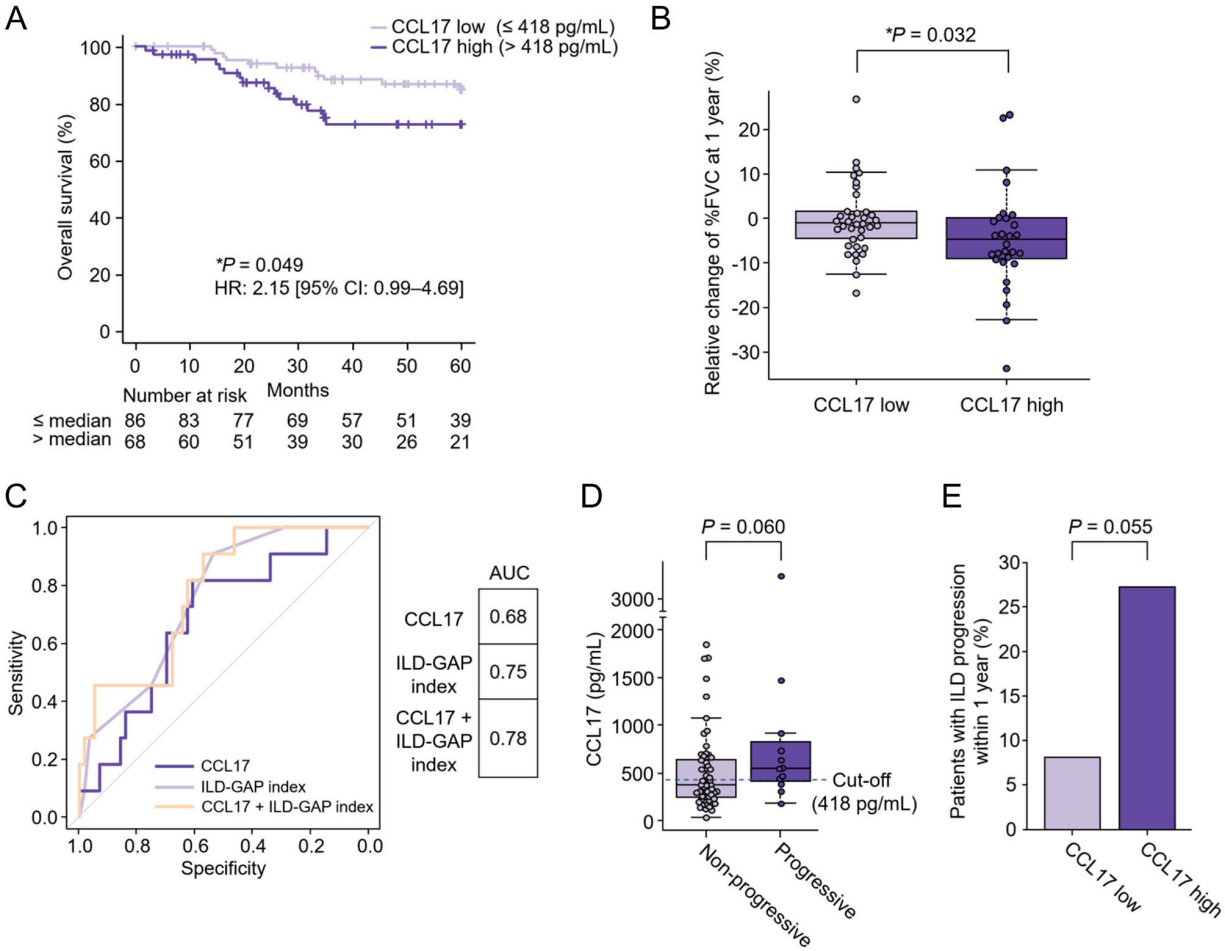
Reproduction of the utility of serum CCL17 levels for predicting non-IPF-ILD progression in the validation cohort. (**A**) Kaplan–Meier curves estimating the probability of overall survival stratified by the serum levels of CCL17 (*n* = 154). (**B**) Relative change of %FVC at 1 year in groups with high (*n* = 68) and low CCL17 levels (*n* = 86). (**C**) Receiver operating characteristic curves for evaluating serum CCL17 and ILD-GAP index as predicting composite outcome (relative decline in %FVC ≥ 10%, acute exacerbation, or death) within a year in 67 ILD cases with complete data. (**D**) Serum CCL17 levels of patients with (*n* = 12) and without ILD progression within 1 year (*n* = 70). (**E**) Percentages of patients with ILD progression within 1 year in groups with high and low CCL17 levels. CCL, C-C motif chemokine ligand; %FVC, percent predicted forced vital capacity; GAP, gender age physiology; HR, hazard ratio; ILD, interstitial lung disease

Consistent with the discovery cohort, univariate analysis by Cox proportional hazards analysis and log-rank tests revealed that the CCL17 high group showed significantly higher mortality than the CCL17 low group (HR 2.15; 95% CI 0.99–4.69; *P* = 0.049 by log-rank test) (Fig. 5A). Moreover, multivariate analysis by Cox proportional hazard regression model identified CCL17 level as an independent prognostic factor from corticosteroid or immunosuppressive agents use and ILD-GAP index (HR 2.42; 95% CI 1.08–5.44; *P* = 0.032) (Table 3). Time-dependent ROC analysis for predicting 3-year survival showed an AUC of 0.63 (95% CI 0.48–0.77) for CCL17 levels (Supplementary Table S4). The relative change of %FVC at 1 year in the CCL17 high group was significantly lower than the CCL17 low group (*P* = 0.032) (Fig. 5B). ROC analysis for evaluating the CCL17 levels as predicting ILD progression within 1 year showed an AUC of 0.68 (95% CI 0.51–0.85). There was no statistical difference between CCL17 levels and the ILD-GAP index (*P* = 0.51) and a combination of CCL17 levels and the ILD-GAP index showed a higher AUC of 0.78 (95% CI 0.64–0.91) (Fig. 5C). There were a trend towards higher serum levels of CCL17 in patients with ILD progression within 1 year than in those without (*P* = 0.060) and a trend towards a higher proportion of patients with ILD progression within 1 year in the CCL17 high group than in the CCL17 low group (27.27% vs. 8.11%; *P* = 0.055) (Fig. 5D and E). This trend was also shown in the subgroup analysis by ILD classification (Supplementary Fig. S5A and B). Thus, the utility of serum CCL17 for predicting non-IPF-ILD progression was also confirmed in the validation cohort.

**Table 3:** Cox proportional hazards analysis of overall survival in patients with complete data for all variables in the validation cohort (*n* = 142)

Variables	Univariate analysis			Multivariate analysis		
	HR	95% CI	*P*	HR	95% CI	*P*
Age (> 71 vs. ≤ 71)	1.16	0.53–2.54	0.71	–	–	–
Sex (male vs. female)	1.46	0.65–3.24	0.36	–	–	–
Smoking history (yes vs. no)	1.30	0.59–2.85	0.52	–	–	–
Corticosteroid or immunosuppressive agents (yes vs. no)	0.64	0.25–1.59	0.33	0.62	0.25–1.57	0.32
%FVC (< median vs. ≥ median)	3.35	1.40–8.04	*0.0068	–	–	–
%DLco (< median vs. ≥ median)	3.00	1.25–7.19	*0.014	–	–	–
ILD-GAP index (> 2 vs. ≤ 2)	3.00	1.37–6.59	*0.0062	3.00	1.36–6.61	*0.0063
CCL17 (> 418 vs. ≤ 418)	2.22	1.00–4.96	0.051	2.42	1.08–5.44	*0.032

CCL17: C-C motif chemokine ligand 17, %DLco: percent predicted diffusing capacity for carbon monoxide, %FVC: percent predicted forced vital capacity, GAP: gender age physiology, HR: hazard ratio, ILD: Interstitial lung disease, 95%CI: 95% confidence interval

### **Increased levels of CCL17 in the lung tissues of patients with PPF**

**Fig. 6:**
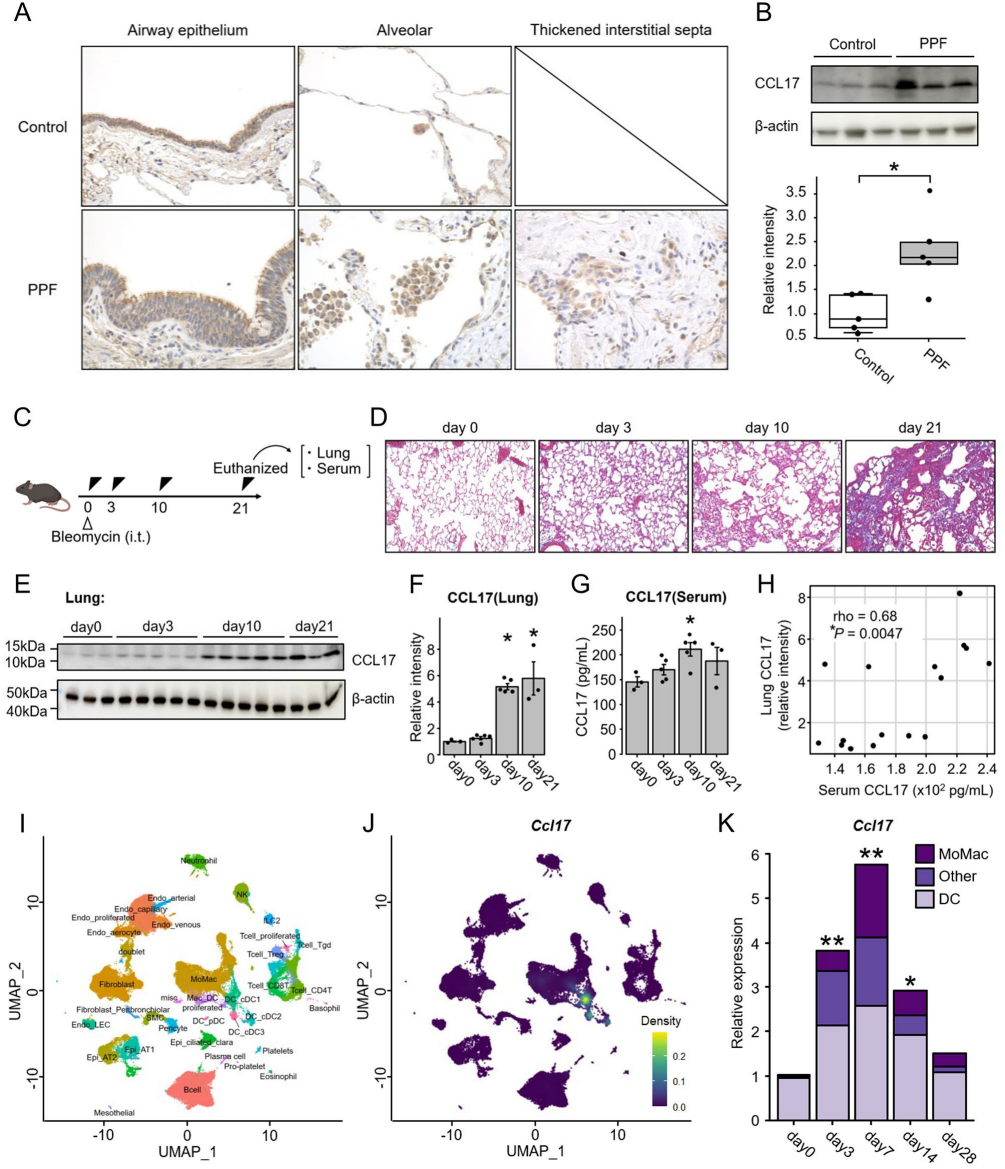
Increased levels of serum CCL17 reflecting an increase in CCL17 in lung tissues during progressive fibrosis. (**A**) A representative image of immunohistochemistry for CCL17 using lung sections from controls and PPF cases. Magnification, 400×. Arrows indicate alveolar macrophages. (**B**) Western blotting for evaluating CCL17 in 10 lung tissue specimens, including 5 PPF surgical specimens and 5 control tissues. A representative blot is shown at the upper panel. (**C**) Schematic representation of the experimental protocol used for the measurement of CCL17 levels of lung tissues and serum from bleomycin-induced pulmonary fibrosis model mice. (**D**) Representative Azan staining of lung tissues. Magnification, 200×. (**E**, **F**) Western blotting analysis of CCL17 in lung tissues and quantification of the levels of CCL17. (**G**) The serum levels of CCL17 measured by an enzyme-linked immunosorbent assay kit. (**E**–**G**) *n* = 3–5 mice per group. (**H**) Spearman correlation between CCL17 levels in lung tissues and those in serum. (**I**) Uniform Manifold Approximation and Projection embedding of single-cell transcriptomes from 77,656 cells from 5 control mice (on day 0) and 20 bleomycin-induced mouse lungs (on days 3, 7, 14, 28, *n* = 5 mice per group) annotated by cell type. (**J**) Density plots of *Ccl17* mRNA expression levels. (**K**) Changes in the expression of *Ccl17* mRNA by pseudobulk analysis. *n* = 5 mice per group. The bars indicate the mean in each of the five mice. (**F**, **G**, **K**) The levels were compared by analysis of variance (ANOVA), and Dunnett’s method was applied to adjust for the ANOVA *P* values. *P < 0.05; **P < 0.01. CCL, C-C motif chemokine ligand; cDC, conventional dendritic cells; MoMac, monocytes and macrophages; PPF, progressive pulmonary fibrosis

In immunohistochemistry analysis, intense CCL17 staining was found in airway epithelial cells and alveolar macrophages. Additionally, CCL17 expression was increased in some thickened interstitial septa in PPF (Fig. 6A and Supplementary Fig. S6). Western blotting analysis using lung tissues showed increased levels of CCL17 in patients with PPF compared with those in controls (Fig. 6B).

### Increased levels of serum CCL17 reflecting an increase in CCL17 in lung tissues during progressive fibrosis

To evaluate the longitudinal expression of CCL17 in lung and serum during the development of fibrosis, we utilized a mouse model of bleomycin-induced pulmonary fibrosis. The extent of fibrosis in the lung tissues was assessed using Azan staining on day 0, day 3, day 10, and day 21 after bleomycin treatment. We found that fibrosis was observed after day 10 of bleomycin treatment, with the strongest fibrosis observed on day 21, as previously reported [17] (Fig. 6C and D). Consistent with those in patients with PPF, CCL17 levels were increased in lung tissues after bleomycin treatment (Fig. 6E and F). Similarly, CCL17 levels were increased in the serum, peaking on day 10 (Fig. 6G). Notably, the CCL17 levels in lung tissues were strongly correlated with those in serum, suggesting that the increased levels of serum CCL17 could reflect an increase in CCL17 in lung tissues (Fig. 6H).

**Fig. 7:**
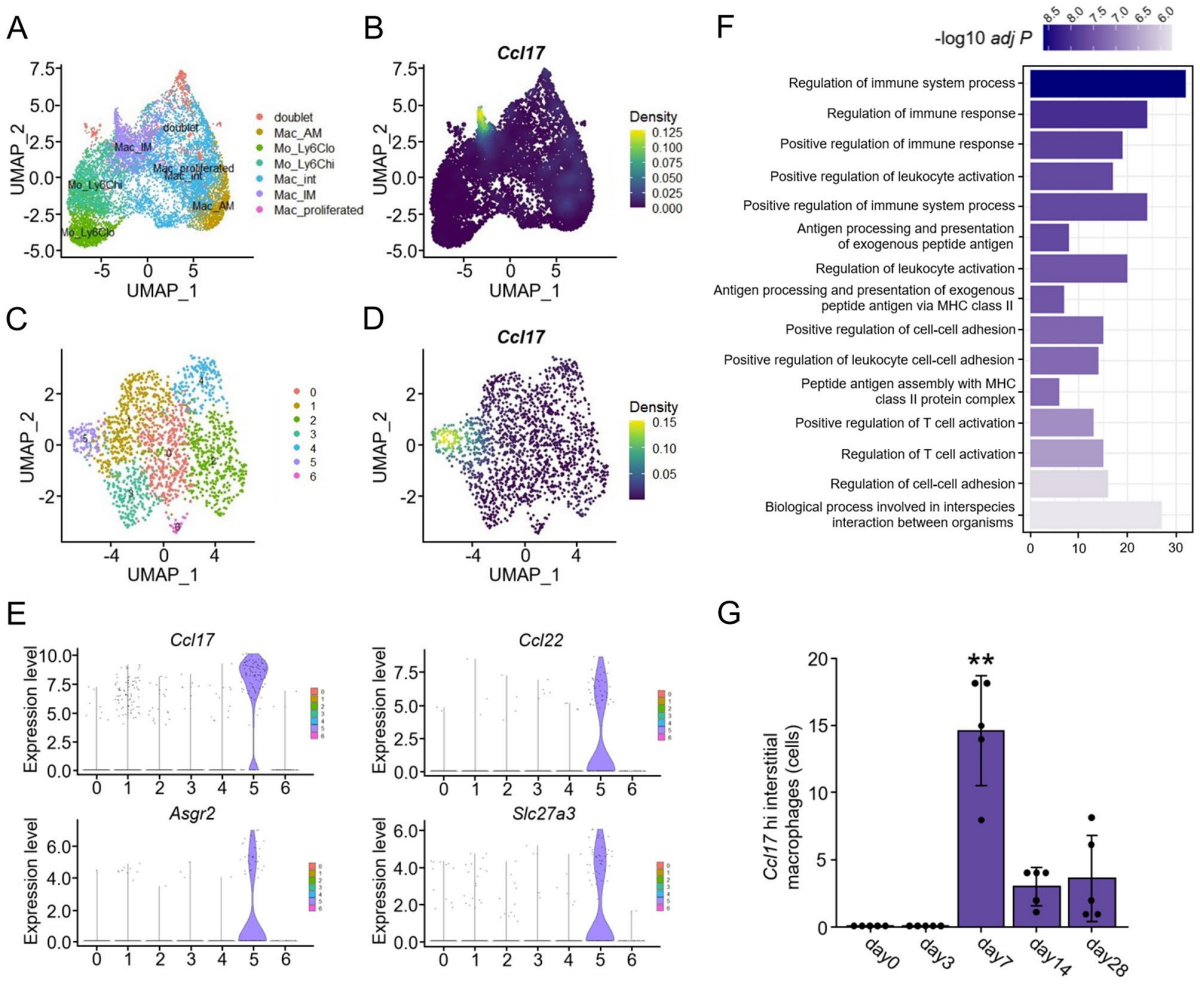
Identification and characterization of the subpopulation with high *Ccl17* expression among monocytes and macrophages. (**A**, **B**) UMAP and density plots describing the distribution of *Ccl17* expressing cells in monocytes and macrophages. Interstitial macrophages show high mRNA expression levels of *Ccl17*. (**C**, **D**) UMAP and density plots describing the distribution of *Ccl17* expressing cells in interstitial macrophages. Cluster 5 shows high mRNA expression levels of *Ccl17*. (**E**) Violin plots of *Ccl17* and representative genes co-expressed with *Ccl17* in cluster 5. (**F**) The 15 most significantly (*P* < 0.05) enriched terms in Gene Ontology biological process in the 100 most significantly co-expressed genes with *Ccl17* in cluster 5. This analysis was performed using a string database. (**G**) Changes in the number of cells in cluster 5. *n* = 5 mice per group. The numbers were compared by analysis of variance (ANOVA), and Dunnett’s method was applied to adjust for the ANOVA *P* values. *P < 0.05; **P < 0.01. Ccl, C-C motif chemokine ligand; Asgr, asialoglycoprotein receptor; Slc27a3, solute carrier family 27 member 3

In addition, we performed single-cell RNA sequencing (scRNA-seq) analysis using the dataset of a mouse model of bleomycin-induced pulmonary fibrosis provided by our previous study [11]. *Ccl17* mRNA was mainly expressed in conventional dendritic cells and part of monocytes and macrophages (Fig. 6I and J). Pseudobulk analysis revealed that the levels of *Ccl17* primarily derived from conventional dendritic cells and monocytes and macrophages elevated after bleomycin administration. *Ccl17* were upregulated during a relatively early phase, suggesting that CCL17 upregulation may reflect profibrotic changes rather than completed fibrosis formation (Fig. 6K). Furthermore, density plots showed that *Ccl17* expression was strong in especially interstitial macrophages among monocytes and macrophages (Fig. 7A and B). Among interstitial macrophages, a subpopulation with particularly strong *Ccl17* expression (*Ccl17* hi Mac_IM) was identified and this population had *Ccl22*, *Asgr2*, and *Slc27a3* as co-expressed genes (Fig. 7C–E and Supplementary Table S5). Gene Ontology biological process analysis of these co-expressed genes revealed enrichment in terms such as the positive regulation of T cell activation (Fig. 7F). Consistent with the results of pseudobulk analysis, the number of *Ccl17* hi Mac_IM increased during a relatively early phase, peaking on day7 (Fig. 7G).

## Discussion

Herein, we measured the serum levels of the 13 candidate biomarkers by CLEIA methods to identify the predictive biomarkers for ILD progression. CCL17 was identified as a predictive biomarker for non-IPF-ILD progression and the high reproducibility of utility was confirmed in another independent cohort. Furthermore, serum CCL17 was an independent prognostic factor from corticosteroid or immunosuppressive agents use and ILD-GAP index. Since our findings were obtained using CLEIA, a clinically applicable assay system, serum CCL17 is expected to have immediate clinical application.

CCL17, as known as Thymus and activation-regulated chemokine, is a CC-chemokine that is induced by interleukin (IL)-4 and IL-13, signature cytokines of type 2 inflammation. It acts as a functional ligand for C-C chemokine receptor type 4 and participates in the trafficking of Th2 cells in eosinophil-associated disorders, including allergic asthma and atopic dermatitis [18, 19]. In Japan, serum CCL17 levels have been commercially measured since 2008 as a biomarker for atopic dermatitis [20, 21]. On the other hand, increasing evidence suggests that a Th2-mediated process including CCL17 plays an important role in the development of pulmonary fibrosis [22–24]. For instance, it has been reported that CCL17 drives fibroblast activation by enhancing tumor growth factor beta (TGF-β)/Smad signaling, and that anti-CCL17 antibodies attenuate fibrosis in a bleomycin-induced pulmonary fibrosis mouse model [23, 24]. However, reports on serum CCL17 as a biomarker for ILD progression remain limited [9, 13]. Our study demonstrated the utility of serum CCL17 as a predictive biomarker for non-IPF-ILD progression in two independent cohorts and further suggested a specific cut-off value of 418 pg/mL. Serum CCL17 levels could enable the stratification of patients at risk of non-IPF-ILD progression, leading to appropriate early therapeutic intervention.

Furthermore, using samples of patients with PPF and model mice, we found that CCL17 levels in the serum have positive correlation with those in the lung tissues. These results suggested that increased CCL17 levels in serum could reflect those in the lung tissues. Serum CCL17 may serve as a liquid biopsy in patients with ILD. In addition, scRNA-seq analysis of a mouse model showed increased levels of *Ccl17* in monocytes and macrophages, as well as conventional dendritic cells during a relatively early phase after bleomycin administration. CCL17 upregulation may reflect profibrotic changes rather than completed fibrosis formation, supporting the serve of serum CCL17 as a predictive biomarker for non-IPF-ILD progression. Considering that CCL17 can induce lung fibrosis [23, 24], stratifying patients by serum CCL17 levels and developing personalized treatments targeting CCL17 for those with high levels would be a worthwhile challenge. The scRNA-seq analysis also suggested the presence of *CcL17* hi interstitial macrophages that specifically co-express genes such as *Ccl22*, *Asgr2*, and *Slc27a3*. This subpopulation, which is considered similar to M2a macrophages because of the co-expression of M2a macrophage markers such as *Il-1r* and *Cd209* [25], may be a potential therapeutic target. Additionally, these results also suggest the possible association between the specifically co-expressed genes such as *Ccl22*, *Asgr2*, and *Slc27a3*, and pulmonary fibrosis.

In our study, the utility of CCL17 for predicting ILD progression differed between IPF and non-IPF-ILD. The neutralizing antibody SAR156597 for IL-4 and IL-13, which directly induce CCL17, reduced the CCL17 levels in the plasma and exhibited significant improvement in the skin scores and a trend toward improved respiratory function in a phase II study of the multiorgan fibrosing disease scleroderma [26]. By contrast, SAR156597 treatment was not effective in a phase II study of IPF [27]. The differences between the type 2 immune responses in IPF and non-IPF-ILD, as suggested in these studies, may have influenced the results of our study. In contrast, it is also possible that the low detection sensitivity due to the relatively small sample size affected our results. There has been a report that increased levels of plasma CCL17 is associated with death in IPF [10], thereby warranting further validation.

Our study also suggested that other biomarkers, such as serum pro SP-B and total POSTN, may be useful biomarkers for predicting ILD progression. SP-B is a member of the protein families SP-A, B, C, and D, which, together with phospholipids, constitute surfactants [28]. While the mature SP-B is too hydrophobic to circulate in the bloodstream, immature SP-B proteins, such as pro SP-B and Cpro SP-B, are less hydrophobic and detectable in the serum [29]. Our previous study suggested that SP-B in serum EVs, which more specifically consisted of its pro-form than SP-B in serum, could serve as a biomarker for predicting ILD progression [11]. In contrast, the study showed no significant association between serum SP-B measured with the commercial ELISA kits and ILD progression [11]. However, the present study has shown an association between pro-form of SP-B in serum measured with our newly developed assay system and ILD progression. Pro-form of SP-B in serum, may be useful for predicting ILD progression, like SP-B in serum EVs. POSTN is a matricellular protein which belongs to the fasciclin family and induced by IL-4 and IL-13 [12, 30]. POSTN in fibroblasts is involved in cross-talk with TGF-β and is implicated in the pathogenesis of pulmonary fibrosis [30, 31]. Serum levels of POSTN have been reported to increase in IPF and other fibrotic ILDs and be associated with declines in DLco and vital capacity [12, 32]. Further validation of these biomarkers is also desirable. In addition, as the combination of CCL17 and total POSTN showed excellent performance for predicting non-IPF-ILD progression in the discovery cohort, developing a combination of multiple biomarkers is expected to more accurately determine the complex pathogenesis of pulmonary fibrosis.

Despite notable advantages, our study has certain limitations. First, this is a single-center cohort study with a relatively small sample size. However, the utility of serum CCL17 for predicting non-IPF-ILD progression was supported by the findings of two independent cohort studies and experiments of human samples and a mouse model. Second, considering the increase of serum CCL17 levels due to eosinophilic disease, we excluded patients with eosinophilic disease or increased blood eosinophil counts from the validation cohort. The utility of serum CCL17 levels in patients with eosinophilic comorbidities remains unclear. Third, the impact of ILD therapeutics such as corticosteroid or immunosuppressive agents on the serum CCL17 levels has not been addressed, thereby warranting further validation.

## Conclusions

Our study has identified serum CCL17 as a clinically measurable biomarker for predicting non-IPF-ILD progression and revealed the dynamics of CCL17 that support its utility as a predictive biomarker. Serum CCL17 levels could enable the stratification of patients at risk of non-IPF-ILD progression, leading to appropriate early therapeutic intervention. Further prospective large-scale studies are needed to corroborate our findings.

## Electronic supplementary material

Below is the link to the electronic supplementary material.

Supplementary Material 1

Supplementary Material 2

Supplementary Material 3

Supplementary Material 4

## Data Availability

The datasets generated and/or analyzed during the current study are available from the corresponding author on reasonable request.

## Abbreviations

ANOVA: Analysis of variance
AUC: Area under the curve
CCL: C-C motif chemokine ligand
CI: Confidence interval
CLEIA: Chemiluminescent enzyme immunoassay
COPD: Chronic obstructive pulmonary disease
CXCL: C-X-C motif chemokine ligand
EV: Extracellular vesicles
GAP: Gender-age-physiology
IL: Interleukin
ILD: Interstitial lung diseases
IPF: Idiopathic pulmonary fibrosis
KL-6: Krebs von den Lungen-6
MMP: Matrix metalloproteinase
Mono POSTN: Monomeric periostin
NSIP: Nonspecific interstitial pneumonia
OS: Overall survival
PPF: Progressive pulmonary fibrosis
ROC: Receiver operating characteristic
SP: Surfactant protein
ScRNA-seq: Single-cell RNA sequencing
TGF-β: Tumor growth factor beta
