# Supplementary material for: Serum C-C motif chemokine ligand 17 as a predictive biomarker for the progression of non-idiopathic pulmonary fibrosis interstitial lung disease
Source: Respir Res. 2025 Apr 23;26:157. doi: 10.1186/s12931-025-03237-2 (PMC12020124; doi:10.1186/s12931-025-03237-2)
Supplement: Supplementary file 1 — Supplementary Material 1 [file 12931_2025_3237_MOESM1_ESM.pdf]

Supplementary Methods

Serum C-C motif chemokine ligand 17 as a predictive biomarker for the progression of interstitial lung disease

Takatoshi Enomoto, Yoshito Takeda, Yuya Shirai, Takehiro Hasegawa, Feng Zhao, Hanna Lunding, Moritz Pohl, Ryuya Edahiro, Shigeyuki Shichino, Takahiro Kawasaki, Hanako Yoshimura, Reina Hara, Saori Amiya, Makoto Yamamoto, Daisuke Nakatsubo, Satoshi Tanizaki, Mana Nakayama, Yoshimi Noda, Takayuki Niitsu, Yuichi Adachi, Mari Tone, Yuko Abe, Maiko Naito, Kentaro Masuhiro, Yujiro Naito, Takayuki Shiroyama, Kotaro Miyake, Shohei Koyama, Kiyoharu Fukushima, Kota Iwahori, Haruhiko Hirata, Izumi Nagatomo, Satoshi Nojima, Masahiro Yanagawa, Yoshikazu Inoue, and Atsushi Kumanogoh

Measurement of serum monomeric and total periostin levels using the newly established HISCL^{TM} reagent

The serum monomeric and total periostin levels were measured using the newly established HISCL^{TM} reagent. The levels were strongly correlated with those measured by previous ELISA methods (**Supplementary Fig. S1**) [1].

Measurement of serum pro SP-B levels using the newly established HISCL^{TM} reagent

In our previous study, the serum levels of SP-B measured with the commercial ELISA kits did not show a significant association with ILD progression [2]. However, it was not clear which SP-B form the ELISA kits were specific to. In the present study, we found that Cpro-form of SP-B was used as the immunogen during antibody production and as the standard in the kits, and that the kits have low reactivity to pro-form of SP-B (**Supplementary Table S1**).

Thus, we established a novel assay system using HISCL^{TM} for quantifying pro-form of SP-B in serum. We confirmed that there is no cross-reactivity with Cpro-form of SP-B (**Supplementary Table S2**).

Immunohistochemistry analysis

Lung tissue samples were obtained from patients without and with progressive pulmonary fibrosis who underwent surgery for suspected lung cancer. Specimens were prepared from areas without tumor lesions. Paraffin-fixed tissues were deparaffinized using xylene and alcohol, incubated with EDTA buffer (pH, 9.0) for antigen retrieval, oxidized using 3% hydrogen peroxide at room temperature for 10 min, and then blocked with 3% bovine serum albumin in PBS at room temperature for 1 h. The slides were incubated with anti-C-C motif chemokine ligand 17 (CCL17) antibodies (1:100; ab182793; Abcam, Cambridge, UK), followed by incubation with horseradish peroxidase-conjugated anti-rabbit (724142; Nichirei Biosciences, Tokyo, Japan) secondary antibody at room temperature for 30 min. Image acquisition was performed using an OLYMPUS-BX51 microscope (Olympus, Tokyo, Japan).

Western blotting analysis

To examine the levels of CCL17 in lung tissues in patients with PPF and bleomycin-induced pulmonary fibrosis model mice compared with those in controls, we performed western blotting. Protein samples were loaded onto NuPAGE 4–12% or 12% Bis-Tris gels (Thermo Fisher Scientific, Waltham, MA, USA). For immunoblot analysis, the gels were electroblotted

onto polyvinylidene difluoride membranes (Bio-Rad, Hercules, CA, USA). Membranes were blocked with Blocking One (Nacalai Tesque, Kyoto, Japan) at room temperature for 30 min, incubated with the specific primary antibody (anti-CCL17 antibody [ab182793; Abcam] or anti-β-actin antibody [#5125; Cell Signaling Technologies, Danvers, MA, USA]), and then incubated with horseradish peroxidase-conjugated anti-rabbit (NA934V; GE Healthcare, Chicago, IL, USA) secondary antibody. Immunoreactive signals were visualized using SuperSignal West Atto Ultimate Sensitivity Maximum Chemiluminescent Substrate (Thermo Fisher Scientific) or Chemi-Lumi One Super (Nacalai Tesque) and detected with an ImageQuant LAS500 system (GE Healthcare, Chicago, IL, USA). Band intensities were quantified using ImageJ software (NIH, version 1.53k). CCL17 levels were normalized to the level of β-actin.

ELISA analysis using commercially available kits

The levels of CCL17 in mice serum were measured using the MCC170 ELISA kits (R&D Systems, Minneapolis, MN, USA).

Bleomycin-induced pulmonary fibrosis mouse model

For western blotting analysis and ELISA analysis, we used 7-week-old male mice (C57BL/6J strain, CLEA Japan, Tokyo, Japan) that were bred at specific pathogen free facilities at Osaka University. These mice were anesthetized with isoflurane and administered a single endotracheal dose of bleomycin (5 mg/kg body weight) on day 0. These procedures were performed in accordance with a previously described method [3]. Bleomycin-naive mice (control) were euthanized on day 0, whereas bleomycin treated mice were euthanized on days 3, 10, and 21 for collecting lung tissues and serum (n = 3–5 mice per group). As a randomization strategy, minimization was performed using the following variables: (i) time of the experiment, (ii) experiment conductor, (iii) equipment, and (iv) animal characteristics (sex, age bracket, weight bracket). T.E. and M.N. were aware of the group allocation at all stages of the experiment.

Single-cell analysis using bleomycin-induced pulmonary fibrosis model mice

We analysed the lung single-cell RNA sequencing (scRNA-seq) data from mice provided by our previous study [2]. The data set comprised five untreated control mice and five mice each on days 3, 7, 14, and 28 after bleomycin treatment. Pseudo-bulk data were constructed using a method described by Mary et al [4].

Statistical analyses of the results of experiments using a mouse model

The CCL17 levels in lung tissues and in serum were compared based on conditions using analysis of variance (ANOVA), and the Dunnett's method was applied to adjust the obtained ANOVA *P* values. The correlation between the CCL17 levels in lung tissues and those in serum was analyzed using Spearman’s correlation analysis. The above statistical analyses were performed using the EZR software, version 1.38. Statistical significance was set at $P < 0.05$.

Supplementary references

1. Ohta S, Okamoto M, Fujimoto K, Sakamoto N, Takahashi K, Yamamoto H, et al. The usefulness of monomeric periostin as a biomarker for idiopathic pulmonary fibrosis. PLoS One. 2017;12:e0174547.
2. Enomoto T, Shirai Y, Takeda Y, Edahiro R, Shichino S, Nakayama M, et al. SFTPB in serum extracellular vesicles as a biomarker of progressive pulmonary fibrosis. JCI Insight. 2024;9.
3. Liu T, De Los Santos FG, Phan SH. The bleomycin model of pulmonary fibrosis. Methods Mol Biol. 2017;1627:27-42.
4. Mary P, Meeta M, Jihe L, William G, Radhika K. hbctraining/scRNA-seq_online: scRNA-seq Lessons from HCBC (first release). Zendo. 2022. https://doi.org/10.5281/zenodo.5826256.
