# Supplementary material for: Serum C-C motif chemokine ligand 17 as a predictive biomarker for the progression of non-idiopathic pulmonary fibrosis interstitial lung disease
Source: Respir Res. 2025 Apr 23;26:157. doi: 10.1186/s12931-025-03237-2 (PMC12020124; doi:10.1186/s12931-025-03237-2)
Supplement: Supplementary file 2 — Supplementary Material 2 [file 12931_2025_3237_MOESM2_ESM.pdf]

# Full unedited gel for Figure 6B

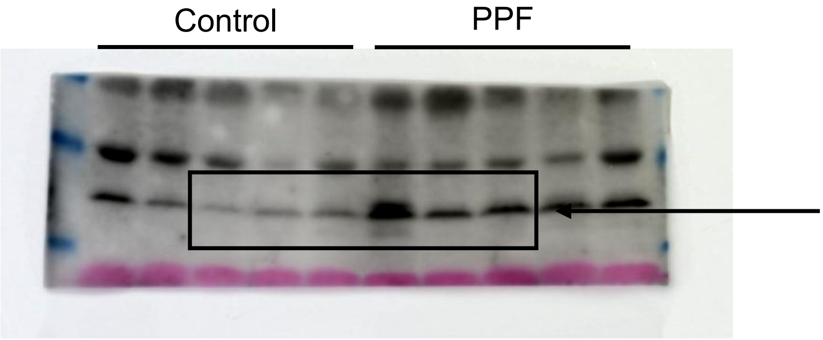


CCL17

Antibody:
ab182793
Abcam

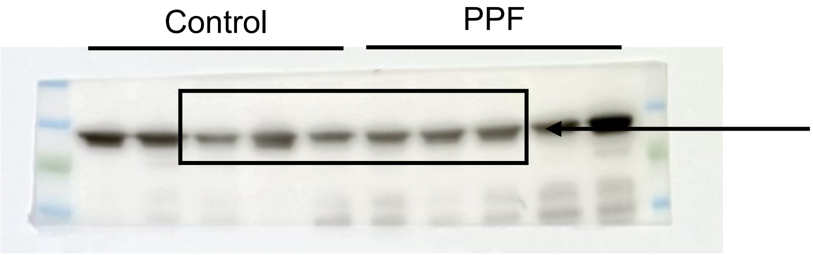


β-actin

Antibody:
#5125
Cell Signaling Technologies

# Full unedited gel for Figure 6D

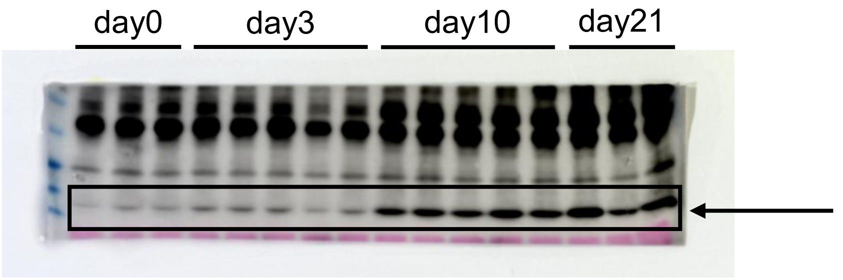


CCL17

Antibody:
ab182793
Abcam

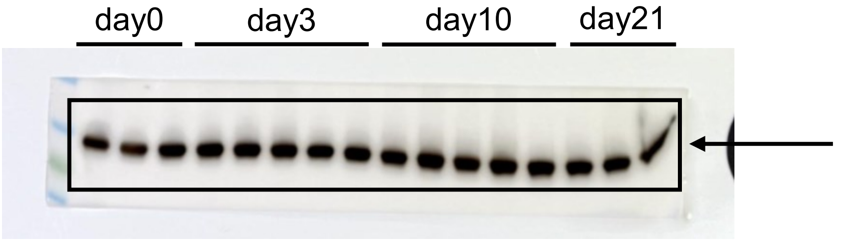


β-actin

Antibody:
#5125
Cell Signaling Technologies
