# Supplementary material for: Serum C-C motif chemokine ligand 17 as a predictive biomarker for the progression of non-idiopathic pulmonary fibrosis interstitial lung disease
Source: Respir Res. 2025 Apr 23;26:157. doi: 10.1186/s12931-025-03237-2 (PMC12020124; doi:10.1186/s12931-025-03237-2)
Supplement: Supplementary file 3 — Supplementary Material 3 [file 12931_2025_3237_MOESM3_ESM.pdf]

Table S1. Low reactivity of commercial ELISA kits to pro-form of SP-B

Pro SP-B input (ng/mL)	OD	ELISA result (ng/mL)	%
3.125	0.004	1.49	47.66
6.25	0.012	1.53	24.49
12.5	0.026	1.61	12.89
25	0.075	1.92	7.66
50	0.143	2.39	4.78

Table S2. No cross-reactivity of the newly established HISCL^{TM} reagent with Cpro-form of SP-B

Cpro SP-B input (ng/mL)	Count	HISCL^{TM} result (ng/mL)	%
0	2.718	0	-
5	2.659	0	0
20	2.781	0	0
80	2.611	0	0
320	2.490	0	0
1280	2.658	0	0

Table S3. Baseline characteristics at the time of blood collection of patients with and without ILD-progression in the discovery cohort

	IPF[†]			Non-IPF-ILD[‡]		
	Non-progressive	Progressive	*P* value	Non-progressive	Progressive	*P* value
No. of subjects	27	17	-	31	11	-
Age (years)	75 (71–78)	69 (61–75)	0.055	73 (67–76)	73 (68–75)	0.79
Sex (male)	25 (93%)	13 (76%)	0.19	15 (48%)	8 (73%)	0.29
Smoking history (yes)	23 (85%)	10 (59%)	0.075	17 (55%)	8 (73%)	0.48
Corticosteroid (yes)	5 (19%)	3 (17.6%)	1.00	13 (42%)	3 (27%)	0.49
Immunosuppressive agents (yes)	1 (4%)	1 (6%)	1.00	8 (26%)	0 (0%)	0.086
Antifibrotic therapy (yes)	8 (30%)	4 (24%)	0.74	0 (0%)	1 (9%)	0.26
Serum biomarkers						
Total POSTN[ǁ] (ng/mL)	17.3 (13.9–27.0)	32.0 (24.1–78.5)	*0.0018	20.5 (13.0–29.8)	45.5 (13.0–65.6)	0.081
CCL17[††] (pg/mL)	506 (373–704)	766 (421–1051)	0.11	387 (290–447)	788 (640–1228)	*3.78x10^{-5}
Pro SP-B[‡‡] (ng/mL)	3239 (2138–5771)	4562 (2400–5026)	0.59	1556 (1078–2566)	3209 (2578–12458)	*0.0029
MMP9[§§] (µg/mL)	12.7 (7.4–24.1)	17.3 (8.8–29.5)	0.46	8.8 (6.5–16.4)	33.6 (15.4–36.4)	*0.0032
Classification of ILD						
INSIP[ǁǁ]	N/A	N/A	N/A	6 (19%)	5 (45%)	0.12
CTD-ILD[†††]	N/A	N/A	N/A	14 (45%)	2 (18%)	0.16
Unclassifiable ILD	N/A	N/A	N/A	10 (32%)	1 (9%)	0.23
FHP[‡‡‡]	N/A	N/A	N/A	1 (3%)	0 (0%)	1.00
PPFE[§§§]	N/A	N/A	N/A	0 (0%)	3 (27%)	*0.014
Pulmonary function tests						
%FVC[ǁǁǁ]	76.0 (62.8–91.5)	64.7 (58.3–81.6)	0.20	83.9 (68.1–97.2)	56.7 (40.5–59.8)	*0.00027
%DLco[††††]	60.4 (54.0–78.2)	48.8 (16.5–56.7)	*0.0041	66.1 (51.1–82.4)	42.0 (35.5–47.9)	*0.0082
ILD-GAP[‡‡‡‡] index			0.15			0.051
0–1	0 (0%)	2 (12%)	-	16 (52%)	0 (0%)	-
2–3	13 (48%)	3 (18%)	-	14 (45%)	5 (45%)	-
4–5	10 (37%)	8 (47%)	-	1 (3%)	2 (18%)	-
6–8	3 (11%)	4 (24%)	-	0 (0%)	0 (0%)	-
N/A	1 (4%)	0 (0%)	-	0 (0%)	4 (36%)	-

Data are presented as the median (interquartile range) or number of patients (percentage). ILD progression was defined as death within a year, acute deterioration within 1 year, or ≥10% decrease in %FVC within 1 year. Differences in progressive group or non-progressive group were assessed by the Mann-Whitney U test or Fisher's exact test. *, $P < 0.05$. [†]idiopathic pulmonary fibrosis; [‡]interstitial lung disease; [§]not available; [ǁ]total periostin; [††]C-C motif chemokine ligand 17; [‡‡]pro-surfactant protein B; [§§]matrix metalloproteinase 9; [ǁǁ]idiopathic nonspecific interstitial pneumonia; [†††]connective tissue disease-interstitial lung disease; [‡‡‡]fibrotic hypersensitivity pneumonitis; [§§§]pleuroparenchymal fibroelastosis; [ǁǁǁ]%forced vital capacity; [††††]%diffusing capacity for carbon monoxide; [‡‡‡‡]gender age physiology.

Table S4. Time-dependent receiver operating characteristic analysis for predicting 3-year survival in patients with complete data for all variables in the validation cohort (n = 142)

Variables	AUC	95% CI
Age	0.46	0.32–0.59
100 – %FVC	0.74	0.59–0.88
100 – %DLco	0.68	0.54–0.81
ILD-GAP index	0.72	0.61–0.82
CCL17	0.63	0.48–0.77
CCL17 + ILD-GAP index	0.73	0.61–0.86

AUC: area under the curve, CCL17: C-C motif chemokine ligand 17, CI: confidence interval, %DLco: percent predicted diffusing capacity for carbon monoxide, %FVC: percent predicted forced vital capacity, GAP: gender-age-physiology, ILD: interstitial lung diseases.

Table S5. The 100 most significantly expressed marker genes in the subcluster of interstitial macrophages with particularly strong *Ccl17* expression

Gene	Average log_2 fold change	Adjusted *P* value
Ccl17	5.74	9.50×10^{-134}
Ccl22	5.62	5.42×10^{-91}
Klrb1b	3.51	6.07×10^{-67}
Asgr2	5.60	1.10×10^{-60}
Mgl2	3.15	5.07×10^{-58}
Dcstamp	3.29	8.66×10^{-57}
Gm44511	2.98	1.58×10^{-56}
Slc27a3	4.58	6.02×10^{-55}
Epcam	5.22	8.29×10^{-53}
P2ry10	3.40	1.67×10^{-46}
Hr	2.96	2.25×10^{-46}
Irf4	3.12	9.39×10^{-46}
Wnt11	3.16	1.73×10^{-45}
Klrk1	4.00	1.26×10^{-44}
Tnip3	2.51	3.30×10^{-42}
Il1r2	2.95	5.68×10^{-42}
Hepacam2	3.28	1.76×10^{-41}
Lmo1	3.39	9.02×10^{-41}
Bcl2a1d	1.51	1.03×10^{-40}
Mmp12	1.65	2.30×10^{-40}
H2-Ab1	1.67	1.28×10^{-39}
AA467197	2.33	6.09×10^{-39}
Plbd1	1.83	4.35×10^{-38}
H2-Eb1	1.62	2.38×10^{-37}
H2-DMb1	1.55	2.18×10^{-35}
Adgrg5	2.91	2.41×10^{-35}
Jak2	2.38	6.29×10^{-35}
Cd74	1.38	1.39×10^{-33}
Syngr2	1.48	2.90×10^{-32}
H2-DMa	1.27	6.37×10^{-32}
H2-Aa	1.39	1.79×10^{-30}
Bhlhe40	1.53	2.06×10^{-30}
Chn2	2.57	4.23×10^{-29}
Rel	1.49	3.35×10^{-28}
Dpp4	3.78	1.84×10^{-27}
Nr4a3	1.84	2.91×10^{-27}
Mmp25	3.52	1.21×10^{-26}
Jaml	2.09	4.10×10^{-26}
Nfkbiz	1.46	2.86×10^{-25}
Malt1	1.47	3.93×10^{-25}
Pxdc1	2.30	5.57×10^{-25}
Tnfaip8	1.22	1.39×10^{-24}
Rpsa	0.71	6.82×10^{-24}
Tbc1d4	2.84	8.55×10^{-24}
Etv3	1.89	1.76×10^{-23}
Olfm1	1.47	4.38×10^{-23}
H2-DMb2	2.71	9.97×10^{-23}
Mir9-3hg	2.62	1.16×10^{-22}

Cd52	0.87	3.47×10^{-22}
Tmsb4x	0.56	4.21×10^{-22}
Napsa	1.48	9.78×10^{-22}
Crip1	1.04	9.86×10^{-22}
Ccdc102a	2.12	3.21×10^{-21}
Gadd45b	1.53	3.38×10^{-21}
Bcl2a1a	1.39	3.58×10^{-21}
Ccr7	3.90	3.59×10^{-21}
Igflr1	2.79	1.35×10^{-20}
Bcl2a1b	0.90	1.97×10^{-20}
Kmo	2.45	4.82×10^{-20}
Clec4b1	2.31	4.85×10^{-20}
Itgax	1.27	5.13×10^{-20}
P2rx5	2.73	1.49×10^{-19}
Gm20503	1.51	2.26×10^{-19}
Cnn2	1.32	2.43×10^{-19}
Srgn	0.70	2.68×10^{-19}
Gng10	1.47	2.87×10^{-19}
Pfkp	1.47	2.91×10^{-19}
Il1b	1.59	7.17×10^{-19}
Rab11fip1	1.23	3.05×10^{-18}
Areg	2.72	3.61×10^{-18}
Myo1g	1.40	5.86×10^{-18}
Lsp1	0.94	7.79×10^{-18}
Cd226	3.30	7.79×10^{-18}
Cd209a	3.93	1.53×10^{-17}
Ear2	1.64	1.92×10^{-17}
Tmem176a	1.20	2.31×10^{-17}
Cfp	1.22	2.72×10^{-17}
Slc2a6	1.98	3.07×10^{-17}
1700025G04Rik	1.53	3.56×10^{-17}
Plet1	3.17	3.95×10^{-17}
Actg1	0.60	4.43×10^{-17}
Nectin1	2.32	6.96×10^{-17}
Gfra2	3.21	9.07×10^{-17}
Atox1	0.82	9.95×10^{-17}
Ramp3	2.23	1.15×10^{-16}
Kdm6b	0.97	1.92×10^{-16}
Gm2a	0.77	4.14×10^{-16}
Il1rl1	2.29	4.41×10^{-16}
F2rl2	2.87	5.52×10^{-16}
Cx3cl1	3.14	6.63×10^{-16}
Sec61b	0.94	6.75×10^{-16}
Macroh2a1	0.93	1.09×10^{-15}
Calcb	2.80	1.19×10^{-15}
Rps11	0.54	1.31×10^{-15}
Rpl23a	0.52	1.34×10^{-15}
Rpl21	0.51	1.41×10^{-15}
Marcksl1	1.23	3.88×10^{-15}
Fyn	1.50	4.77×10^{-15}
Snhg15	1.44	6.38×10^{-15}
Traf1	1.92	1.18×10^{-14}
