# Supplementary material for: Serum C-C motif chemokine ligand 17 as a predictive biomarker for the progression of non-idiopathic pulmonary fibrosis interstitial lung disease
Source: Respir Res. 2025 Apr 23;26:157. doi: 10.1186/s12931-025-03237-2 (PMC12020124; doi:10.1186/s12931-025-03237-2)
Supplement: Supplementary file 4 — Supplementary Material 4 [file 12931_2025_3237_MOESM4_ESM.pdf]

Fig. S1

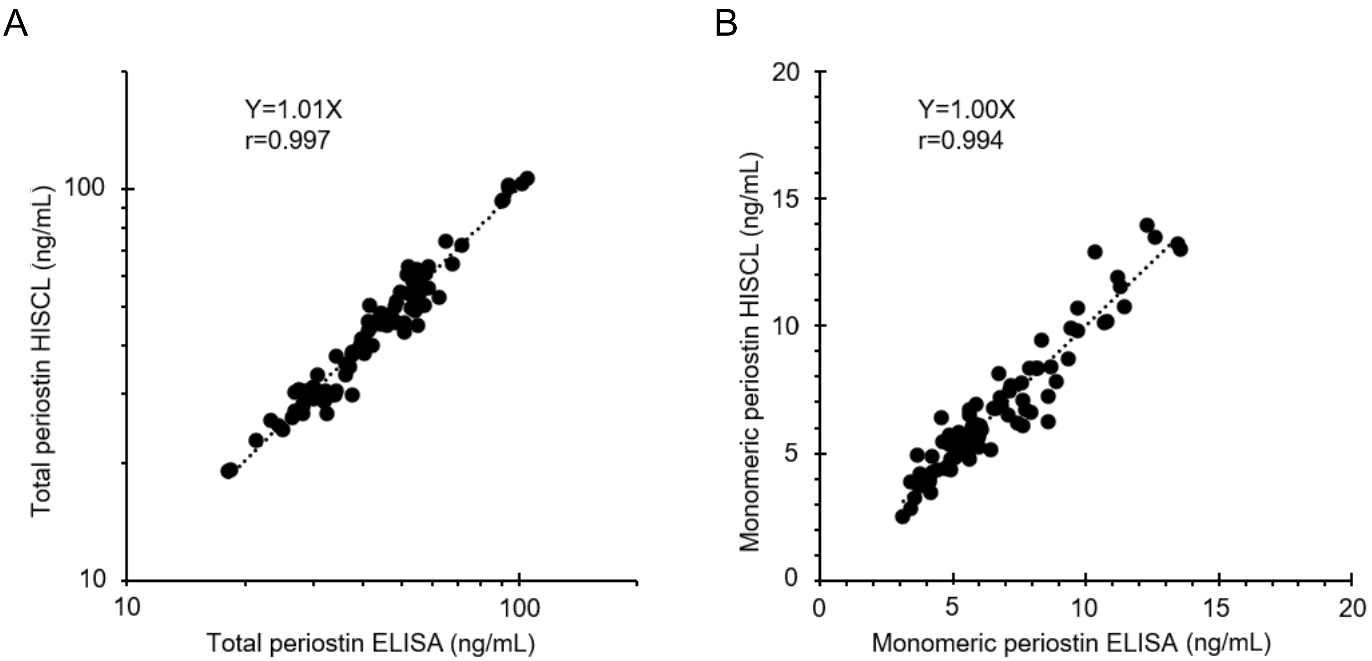

Fig. S1 Correlations between serum levels measured by CLEIA methods using newly established HISCL^{TM} reagent and previously reported ELISA methods for (A) total periostin and (B) monomeric periostin.

Extremely strong correlations were found between serum levels measured by two measurement methods. Antibodies (SS18A ×SS17B) were used for detecting total periostin in both the ELISA and HISCLTM reagent, while antibodies (SS20A and SS19D) were utilized for detecting monomeric periostin. The correlation coefficient was calculated based on the Pearson correlation coefficient.

Fig. S2

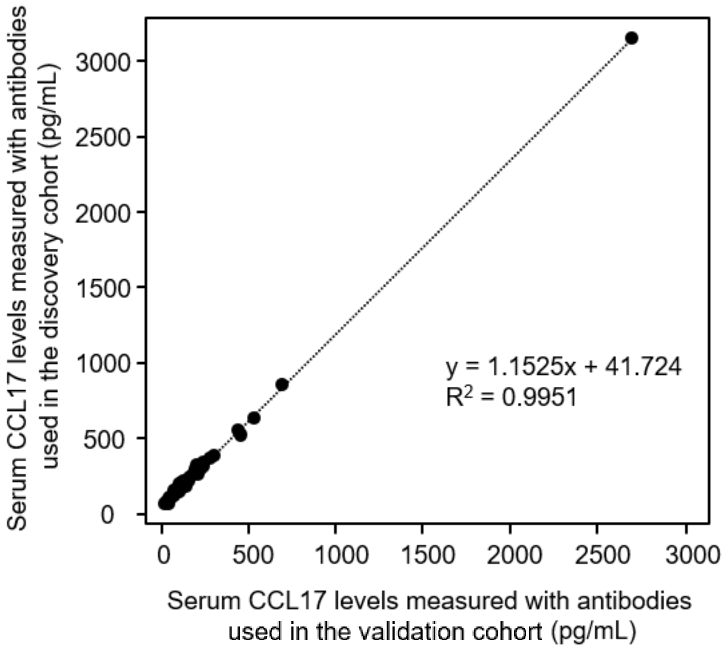

Fig. S2 Correlations between serum CCL17 levels measured by the antibodies used in the discovery cohort and those used in the validation cohort.
Extremely strong correlation was found between serum levels measured by the two measurement methods. Un-linkable anonymized serum and plasma samples for stability tests were provided from MEDILYS Laborgesellschaft mbH of Asklepios Klinik Altona. Linear regression was used to calculate the correlation.

Fig. S3

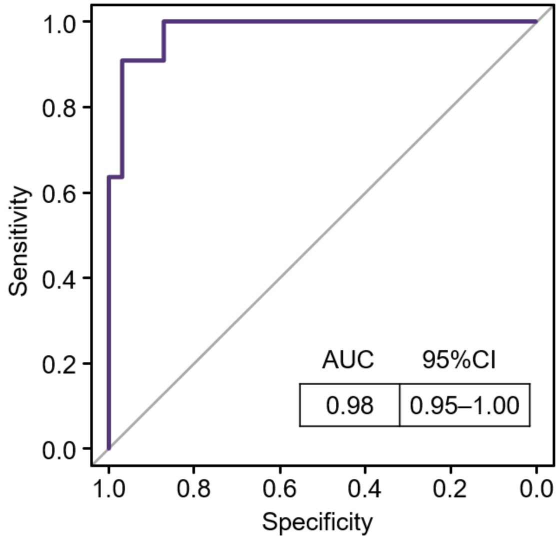

Fig. S3 ROC curves for evaluating the combination of CCL17 and total POSTN for predicting composite outcome (relative decline in %FVC ≥ 10%, acute exacerbation, or death) within a year in 42 evaluable non-IPF-ILD cases in the discovery cohort.

Fig. S4

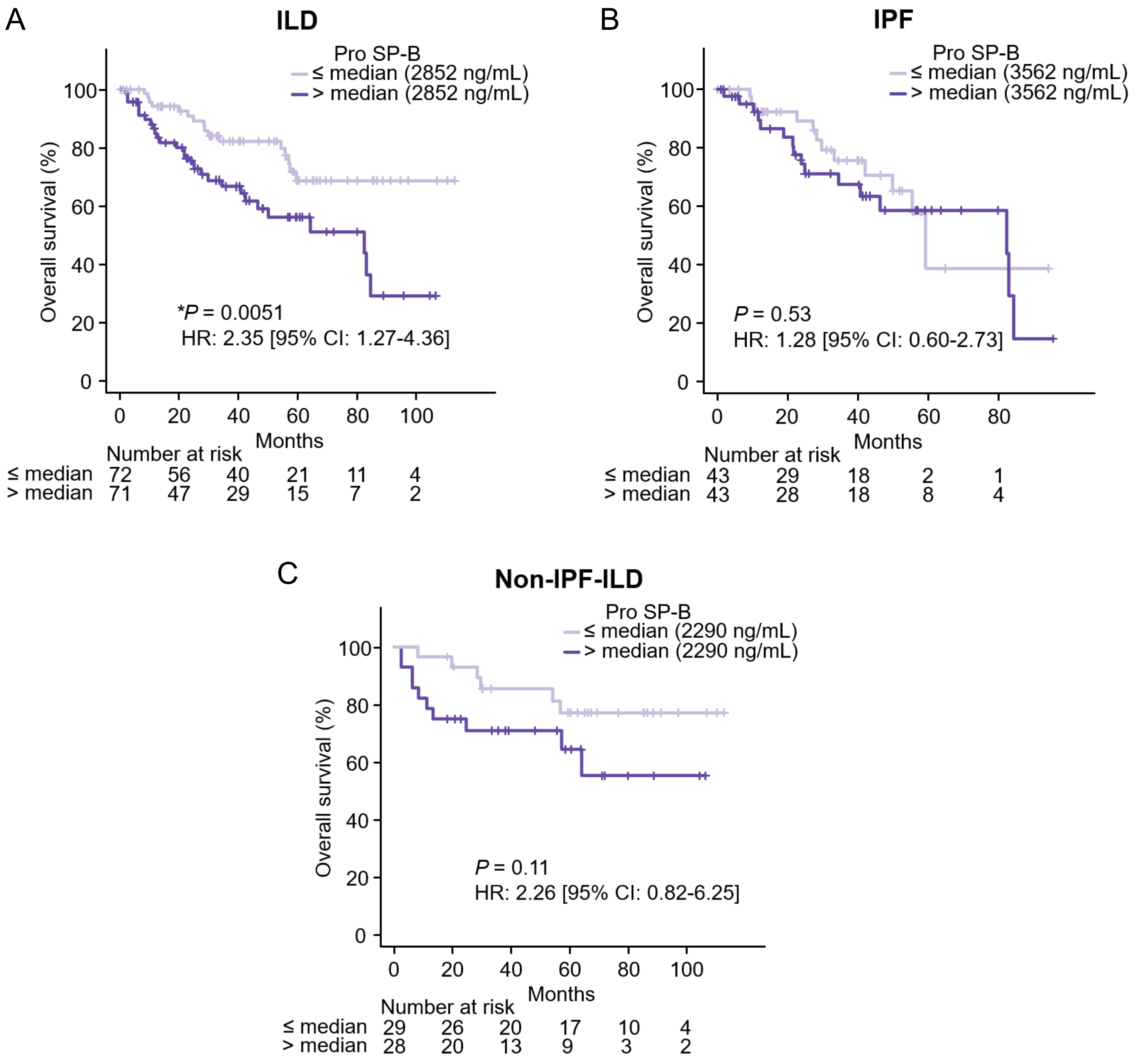

Fig. S4 Kaplan–Meier curves estimating the probability of overall survival (OS) stratified by the serum levels of pro SP-B in (A) 143 patients with ILD, (B) 86 patients with IPF, and (C) 57 patients with non-IPF-ILD.

Fig. S5

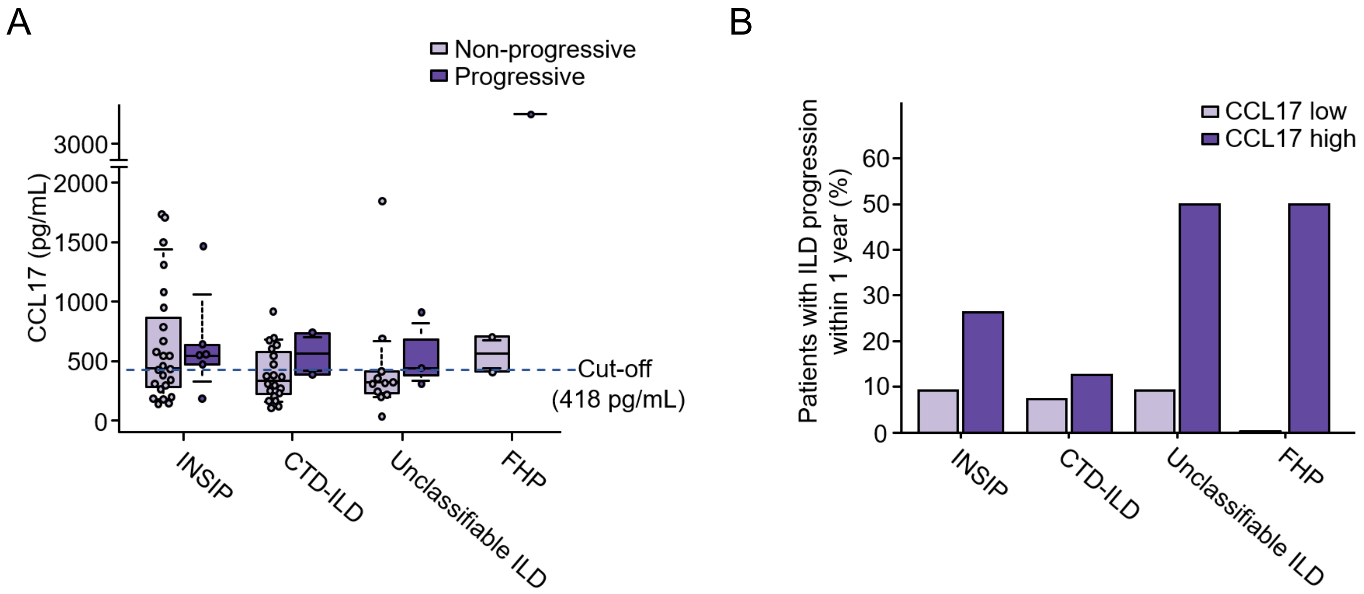

Fig. S5 Subgroup analysis of the validation cohort by ILD classification.
(A) Serum CCL17 levels of patients with and without ILD progression within 1 year for each disease. (B) Percentages of patients with ILD progression within 1 year in groups with high and low CCL17 levels for each disease.

Fig. S6

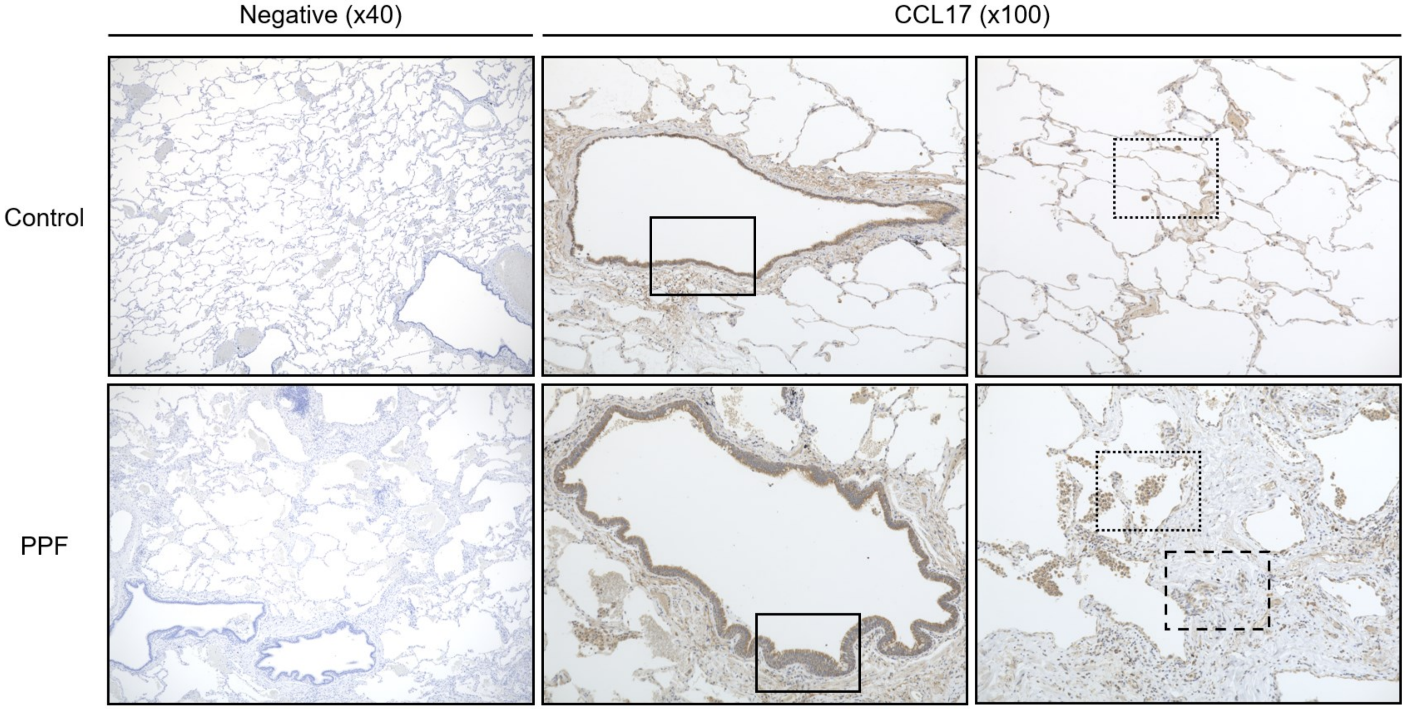

Fig. S6 A representative image of immunohistochemistry for CCL17 using lung sections from controls and progressive pulmonary fibrosis (PPF) cases.
The box, short dotted box, and wide dotted box areas indicate airway epithelium, alveolar, and thickened interstitial septa region in Figure 6A, respectively.
